# Short-term action is key for gigaton-scale Direct Air Capture by 2050

**DOI:** 10.1038/s41467-026-72691-3

**Published:** 2026-05-09

**Authors:** Tatjana Zurbriggen, Nicoletta Brazzola, Adrian Odenweller, Falko Ueckerdt, Joeri Rogelj

**Affiliations:** 1 Institute for Environmental Decisions (IED), ETH Zürich, Zurich, Switzerland; 2 Earth Sciences Department, University of Oxford, Oxford, UK; 3 German Institute for International and Security Affairs (SWP), Berlin, Germany; 4 Potsdam Institute for Climate Impact (PIK), Potsdam, Germany; 5 Interdisciplinary Transformation University Austria, Linz, Austria; 6 Centre for Environmental Policy (CEP) and Grantham Institute – Climate Change and Environment, Imperial College London, London, UK; 7 Energy, Climate and Environment Program, International Institute for Applied Systems Analysis (IIASA), Laxenburg, Austria

**Keywords:** Climate-change mitigation, Climate-change policy

## Abstract

Direct Air Capture (DAC) is widely considered essential for achieving net-zero and net-negative emissions, yet its potential to scale to climate-relevant levels remains uncertain. Here we show that the future deployment of DAC depends primarily on early capacity expansion and growth dynamics rather than on long-term demand targets alone. Using a probabilistic technology diffusion model informed by historical analog technologies and uncertain future demand, we explore a wide range of possible global DAC deployment pathways to 2050. We find that if DAC follows growth trajectories similar to ammonia synthesis technologies and liquefied natural gas, deployment is likely to remain at the megaton scale by mid-century. However, gigaton-scale deployment becomes plausible under rapid growth and strong early policy support. Our results identify short-term capacity expansion as the most effective lever for accelerating DAC deployment and highlight the critical importance of timely policy action to avoid overreliance on future large-scale carbon removal.

## Abstract (web summary)

This study models future Direct Air Capture deployment pathways to 2050, finding that early capacity expansion matter more than long-term demand targets, with gigaton-scale removal only plausible under rapid growth scenarios.

## Introduction

Direct Air Capture (DAC) is a promising family of technologies to tackle climate change. DAC encompasses different technologies^1^, with liquid solvent, solid sorbent, and Calcium oxide (CaO) ambient weathering DAC currently being the dominant designs^1–3^. Combined with CO₂ storage (as Direct Air Carbon Capture and Storage, DACCS), DAC enables long-term carbon dioxide removal (CDR). Unlike other land-based CDR methods, DAC operates with fewer biophysical constraints and possibly lower sustainability trade-offs^4,5^, while offering great flexibility in the choice of the location of its deployment^6^. DACCS could therefore play an important role to counterbalance residual emissions at net-zero and, beyond that, to deliver net-negative emissions^7,8^. Moreover, DAC can also provide CO₂ feedstock to produce synthetic fuels, chemicals, and materials, broadening its application across sectors^9,10^. Although air-captured CO₂ currently remains less cost-competitive than biogenic or point-source CO₂, its strategic value could increase as systems approach net-zero emissions and other feedstock sources become constrained^11,12^. As such, DAC is rising as a promising solution to abate unavoidable residual emissions and achieve net-negative emissions both in modeling^7,8^ and policymaking^13,14^.

Despite its potential, current deployment of DAC remains critically low, with ~27 small-scale plants operational globally, primarily in Europe and North America^15^. Nonetheless, many industry actors have communicated the goal of scaling DAC to the gigaton-scale by 2050^16^, which implies an increase in capacity by 100,000 times from the 2024 annual capture rate of 0.01 MtCO₂ yr⁻¹^17,18^. The promise of gigaton-scale DAC by 2050, unless underpinned by rigorous assessments demonstrating their feasibility, can create unwarranted reliance on large-scale DAC availability to meet net-zero and the Paris climate targets, which has raised concerned of mitigation deterrence^19,20^. This underscores the urgent need for clarity on the scale-up potential of DAC. In particular, assessments of feasible DAC capacity by mid-century and the potential of different policy levers in scaling up this technology are essential to manage expectations and avoid over-reliance on infeasible DAC rates to achieve our climate targets.

Prior literature^21–23^ has begun to address the question of DAC scale-up feasibility, with some studies drawing on technological analogs to bound plausible growth trajectories. Edwards et al.^22^ combined empirical data on historical analogs and early adoption indicators with an integrated assessment model and found that the feasibility space for DACCS is large but highly analog-dependent. Roberts and Nemet^21^ deep-dived into the suitability of a single analog for liquid DAC, ammonia synthesis, finding that reaching fast growth would require aggressive state and industrial support. Unlike previous work on the diffusion of green hydrogen^24^ and of sustainable aviation fuels^25^, previous assessments of DAC diffusion have not fully accounted for the deep uncertainties surrounding DAC’s initial capacity and long-term demand within a single, transparent probabilistic diffusion framework and have neglected the role of policy levers.

In this work, we tackle this gap by investigating possible growth pathways for DAC, considering the large uncertainties associated with DAC growth rates, initial capacity, and future demand. Our study is framed explicitly as a what-if experiment: if DAC diffusion were to approximate empirically observed logistic growth trajectories, what capacity ranges could plausibly result by mid-century? To explore this issue, we adapt a parsimonious, state-of-the-art probabilistic logistic diffusion model developed by Odenweller et al.^24^ for the case of future DAC deployment. Our model is deliberately simple and does not capture the full technological, economic, or policy complexity of DAC deployment, as it is not based on any techno-economic characteristics nor on an assessment of climate policies, markets, and investment choices which may affect DAC diffusion. Our stylized approach offers, however, advantages: it enables transparent exploration of uncertainty, avoids common pitfalls of statistical overfitting for novel energy technologies^26^, and builds on state-of-the-art insights from observed diffusion patterns of past technologies. The model is informed by an extensive database of planned DAC plants, by long-term DAC demand that is randomly sampled from a broad, uniform range, mirroring current uncertainties on the future role of DAC, and by growth rates of three technological analogs, namely ammonia synthesis, liquefied natural gas (LNG), and wind power. In addition to exploring the uncertainty associated with the growth rates of different technological analogs, we model a set of scenarios that mimic cases with enhanced policy support, with the impact of three key policy levers explicitly explored: (i) a policy push fostering short-term DAC capacity in 2030, (ii) the creation of credibility in long-term DAC requirements through an extended demand-pull anticipation, and (iii) the introduction of a higher minimum long-term demand for DAC than in the base case, which we model without enhanced policy support (see Methods). An overview of the uncertain variables can be found in Table 1, while a detailed discussion of the enhanced policy case can be found in the Methods and in Supplementary Method 4.

**Table 1:** Overview of the key uncertain parameters used in this study

Uncertain parameters	Definition	Values (base case)	Values (enhanced policy case)
Initial capacity in 2030	Reflects the transition from formative to growth phase (Supplementary Fig. 1). Our base case (Fig. 1) is based on a truncated normal distribution of empirical direct air capture (DAC) capacity data (Supplementary Table 2); our case with enhanced policy support (Fig. 2a) modeled a significant capacity acceleration (10× increase) driven by political interventions (Fig. 2b).	Mean 8.8 (Truncated min: 4.8) MtCO₂ yr⁻¹	10× base case
Emergence growth rate	Captures maximum annual growth in the post-formative phase (Supplementary Fig. 1). We used ammonia synthesis (~11% yr⁻¹) as the ‘middle-of-the-road’ scenario, wind energy (~20% yr⁻¹) as optimistic, and liquified natural gas (LNG) (~4% yr⁻¹) as pessimistic sensitivity scenarios (Figs. 1a–c and 2a).	LNG: 3.8 (0–6.8) % yr⁻¹ Ammonia: 11(0–25) % yr⁻¹ Wind: 20 (0–42) % yr⁻¹	
Anticipation of direct air capture (DAC) demand	Accounts for investor confidence in DAC’s long-term market potential. Our base case (Fig. 1) assumed an anticipation of 5 years; our case with enhanced policy support (Fig. 2a) extended this to 15 years, reflecting enhanced regulatory certainty regarding long-term DAC requirements (Fig. 2c).	5 years	15 years
Long-term DAC demand	Generated by uniform distributions of potential DAC demand by 2050. Our base case (Fig. 1) ranged from 0 to 5.1 GtCO₂ yr⁻¹ to represent the full range of uncertainty on the potential role of DAC, both as carbon dioxide removal method and to deliver net-zero CO_2_ feedstock; our enhanced policy case (Fig. 2a) represents the 65% upper end of the initial distribution (from 1.79 to 5.1 GtCO₂ yr⁻¹), reflecting improved market conditions driven by policy (Fig. 2d).	0–5.1 GtCO₂ yr⁻¹	1.79–5.1 GtCO₂ yr⁻¹

For further explanations see Supplementary Method 2–6.

## Results

### Uncertainty in DAC deployment emerging from historical analogs

**Fig. 1:**
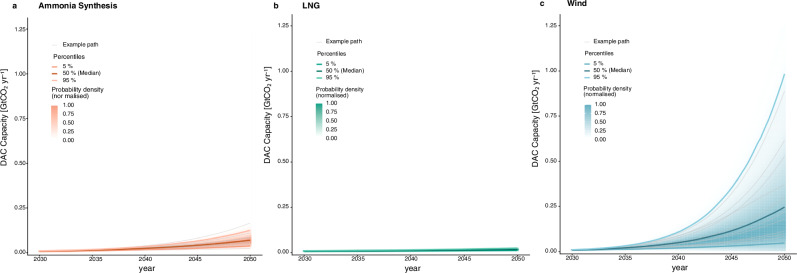
Probability feasibility space for achieving global direct air capture (DAC) deployment by 2050 in scenarios without enhanced policy support (‘base case’). Dashed lines represent the median of the feasibility space, the color shading indicates the annual probability density (determined from the uncertainty propagation of the initial capacity in 2030 and the emergence growth rate), and gray lines show example growth paths, representing the broad spectrum of possible outcomes. **a** Modeled DAC diffusion based on historical growth rates of ammonia synthesis; **b** Modeled DAC diffusion based on historical growth rates of liquified natural gas (LNG); **c** Modeled DAC diffusion based on historical growth rates of wind.

Figure 1 illustrates the wide range of DAC deployment outcomes that emerge from uncertainty in the choice of technological analog, even before considering enhanced policy support. To span a broad range of uncertainty, we selected three technological analogs that share technology similarities with different types of DAC^2,21,27^ and that diverge in their historical growth rates. First, we selected LNG as the lower boundary of technological analogs, based on the identification by Roberts and Nemet^21^ as a rather conservative analog for DAC. Then, we adopted ammonia synthesis as a ‘middle-of-the-road’ analog. While high-temperature (liquid solvent) DAC was found to share technological similarities with ammonia synthesis technologies, the analogy between ammonia synthesis and low-temperature (solid sorbent) DAC is unjustified. Therefore, we also include wind energy not only as an optimistic growth benchmark but also as a closer analog for solid sorbent DAC, due to its higher potential for modularity and mass manufacturing^27^.

Across all base-case scenarios, the achieved DAC capacity by 2050 substantially varies depending on the assumption of which technology DAC could be analogous to. If DAC deployment follows a logistic diffusion curve, the uncertain parameters correspond to the base case (Table 1), and DAC growth would resemble that of ammonia synthesis (Fig. 1a), DAC deployment remains limited through mid-century. In this scenario, DAC diffusion only reaches ~70 MtCO₂ yr⁻¹ by 2050 in the median path. Even at the upper end of the distribution, represented by the 95th percentile, DAC deployment reaches a maximum of ~125 MtCO₂ yr⁻¹ by mid-century. This outcome reflects the relatively modest historical growth rates of ammonia synthesis, which, combined with low 2030 planned DAC capacity and uncertainty around 2050 DAC demand, hampers the fast diffusion of DAC. Assuming growth rates of DAC comparable to those historically observed for LNG leads to even more constrained outcomes (Fig. 1b). Median DAC capacity by 2050 would then only reach roughly 20 MtCO₂ yr⁻¹ by 2050. This pessimistic case highlights that if DAC diffusion were to follow slow-moving, capital-intensive energy infrastructure trajectories, its contribution to climate mitigation would remain marginal for several decades. The most optimistic base-case outcomes emerge if we assumed that the fast growth historically observed for wind power would apply (Fig. 1 c). In that scenario, DAC would in median reach ~250 MtCO₂ yr⁻¹ by 2050, with the gigaton-scale only reached in the 95th percentile of the Monte Carlo runs. These findings illustrate the magnitude of the challenge associated with achieving a global DAC capacity of one gigaton by 2050. If DAC deployment follows the growth trajectories of technological analogs such as ammonia synthesis or LNG, reaching the gigaton scale appears unlikely, while even under optimistic growth assumptions it emerges only in model realizations characterized by particularly favorable initial capacity and long-term demand.

### The impact of policy levers on DAC deployment

**Fig. 2:**
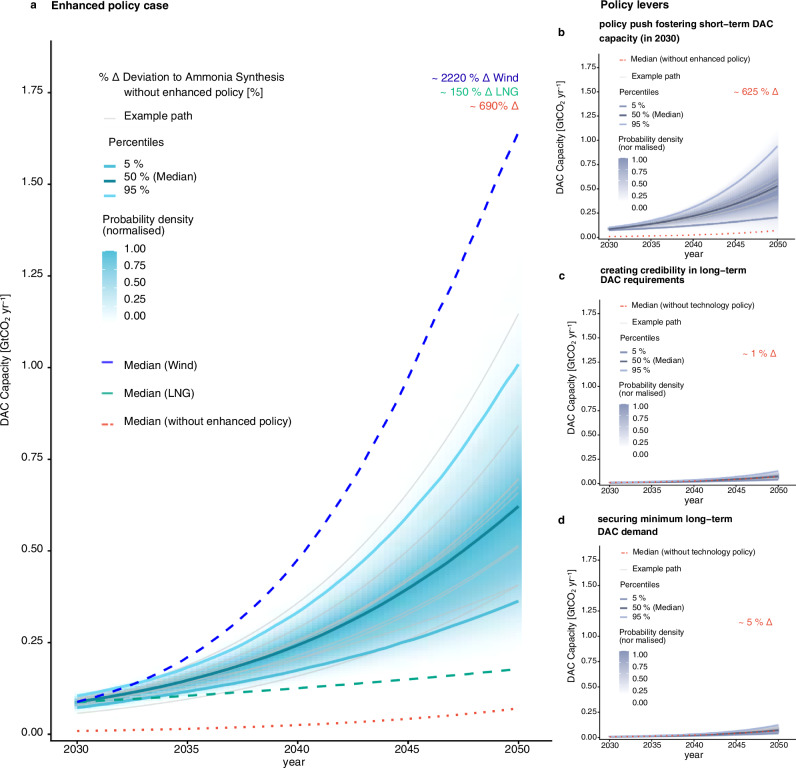
Probability feasibility space for achieving global direct air capture (DAC) deployment by 2050 under enhanced policy scenarios (‘enhanced policy case’). **a** Probability feasibility space for achieving global DAC deployment by 2050 under enhanced policy scenarios with all three policy levers. **b**–**d** Probability feasibility space for achieving global DAC deployment by 2050 under individual policy levers: **b** a policy push to accelerate initial DAC capacity by 2030, **c** measures to establish credible long-term DAC demand, and **d** policies to secure minimum long-term demand. The color shading indicates the annual probability density (determined from the uncertainty propagation of the initial capacity in 2030 and the emergence growth rate), with gray lines showing example growth paths, representing the broad spectrum of possible outcomes. The deviation to the scenarios without enhanced policy support (% Δ) was rounded to the nearest 5th or 10th for B and all plots in (**b**–**d**).

Figure 2a shows how enhanced policy support alters DAC deployment trajectories across the same set of technological analogs. Compared to the base case, enhanced policy consistently shifts the distribution of outcomes upward, increasing both median and upper-percentile capacities by approximately an order of magnitude. Nevertheless, the influence of growth rates remains dominant. Even with a mix of enhanced policy but following the growth rates of ammonia synthesis, DAC capacity improved, reaching ~600 MtCO₂ yr⁻¹ for the median path, but only could reach ~1 GtCO₂ yr⁻¹ for the 95th percentile. If DAC proved to achieve more optimistic growth rates, modeled after wind energy, it could achieve ~1.6 GtCO₂ yr⁻¹. In this case, gigaton-scale DAC becomes the central outcome rather than a tail event. However, this result hinges on the assumption that DAC can replicate the rapid diffusion patterns of modular, mass-manufactured energy technologies, which may not hold for all DAC designs. In contrast, pessimistic growth, modeled after LNG diffusion, resulted in ~180 MtCO₂ yr⁻¹, lower than the case modeled after the growth rates of ammonia synthesis. This reinforces the conclusion that policy support cannot fully compensate for intrinsically slow growth dynamics.

To disentangle the relative importance of individual policy levers, Fig. 2b–d examine their effects in isolation. A short-term capacity push that increases DAC deployment by 2030 emerges as the most influential intervention (Fig. 2b). This early boost raises the median 2050 capacity by more than ~600%, enabling DAC to reach ~500 MtCO₂ yr⁻¹ by 2050 even without additional demand-pull measures. In contrast, policies aimed at strengthening long-term demand credibility without increasing near-term deployment have only marginal effects (~1% and ~5%) (Fig. 2c, d). Without the mentioned policy push, DAC could not exit its formative phase (Supplementary Fig. 1) or achieve meaningful capacity by 2050. Regional findings for Europe and North America showed similar trends (Supplementary Figs. 8, 9).

### Long-term drivers of DAC deployment rates

**Fig. 3:**
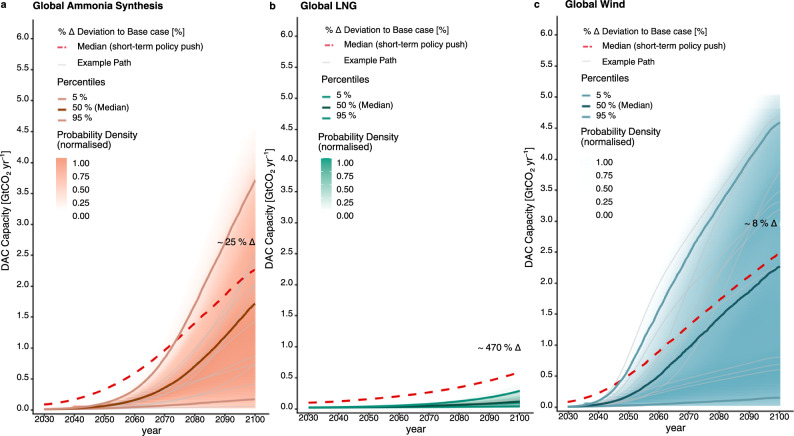
Global direct air capture (DAC) diffusion feasibility space until 2100. DAC deployment until 2100 under growth rates of **a** ammonia synthesis, **b** liquified natural gas (LNG) and **c** Wind on the global level. The red dotted line shows the mean of the respective scenario with short-term policy push.

Extending the time horizon to 2100 reveals a shift in the relative importance of early capacity boosts (Fig. 3). Over longer timescales, differences in growth rates heavily dominate outcomes, while the influence of early capacity pushes diminishes as diffusion trajectories asymptotically approach their saturation levels. Under ammonia-synthesis-like growth (Fig. 3a), median DAC capacity reaches ~1.7 GtCO₂ yr⁻¹ by 2100, with the 95th percentile approaching 4 GtCO₂ yr⁻¹. Under wind-like growth (Fig. 3c), median capacity exceeds 2 GtCO₂ yr⁻¹, with upper-percentile outcomes nearing 4.7 GtCO₂ yr⁻¹. In these cases, early capacity pushes primarily affect cumulative removals rather than end-of-century capacity levels. By contrast, under pessimistic growth assumptions analogous to LNG diffusion (Fig. 3b), DAC deployment stagnates well below climate-relevant levels even by 2100, with median capacity remaining below 200 MtCO₂ yr⁻¹. In this case, early capacity pushes remain important even in the long run, enabling DAC to reach ~500 GtCO₂ yr⁻¹ by 2100. This finding highlights that early policy interventions are most consequential when intrinsic growth rates are low, whereas fast-growing technologies eventually overcome early disadvantages given sufficient time.

## Discussion

Our results demonstrate that mid-century DAC capacity is constrained primarily by growth dynamics and early deployment rather than by long-term demand targets alone. Achieving gigaton-scale DAC by 2050 requires a combination of rapid diffusion rates comparable to those of fast-scaling energy technologies and strong short-term policy support that accelerates early deployment. Absent these conditions, DAC is unlikely to reach scales commensurate with ambitious climate targets by mid-century, even under optimistic assumptions about future demand.

Because the emission gap is increasingly widening^28,29^, reaching the Paris climate goals will likely require multiple gigatons of carbon removal at net zero (7–9 GtCO_2_ yr⁻¹ according to the State of CDR report^30^). Beyond 2050, carbon removal may be deployed for large-scale net-negative emissions, which are likely to be needed to return from overshoots of the 1.5–2 °C target^31^. The extent and pace of the remediation for these overshoots will also depend on the capacity of DAC by mid-century, since other cheaper CDR methods may have already hit scalability barriers in the first half of the 21st Century. Our analysis sheds light on the complex challenge of scaling up DAC such that it could meaningfully contribute to ambitious climate targets. If DAC deployment were to follow the empirical diffusion patterns of analogous technologies, then achieving gigaton-scale DAC by mid-century would require both ambitious growth rates and enhanced policy support. Even with higher initial capacity, better anticipation of demand, and a more narrowly defined long-term DAC target, if DAC were to follow the growth rates of ammonia synthesis (~11% yr⁻¹), in median it would not reach the gigaton-scale. Only highly optimistic scenarios, assuming growth rates as fast as historically observed for wind energy (~20% yr⁻¹) and incorporating different policy levers, could lead to up to 1.5 GtCO_2_ by 2050. Conversely, pessimistic trajectories, with growth rates comparable to those of LNG (~4% yr⁻¹), lead to only about 100 MtCO_2_ DAC by 2050 even if different policy levers apply (Fig. 2a).

Our findings align with prior studies^1,22,32^ emphasizing that DAC would need to follow the growth rates of fast-diffusing technologies, such as photovoltaics and nuclear power, to achieve gigaton-scale deployment by mid-century. Modular and low-complexity technologies like photovoltaics have historically scaled more rapidly as modularity enables iterative learning-by-doing, mass production and cost reductions that spur further growth^27,33^. This challenge is not unique to DAC, as it was shown to apply also to other novel technologies such as green hydrogen^24^. However, our approach departs from earlier DAC diffusion studies^21,22,32^ in three important ways. First, unlike studies that rely on linearly extrapolated analog growth rates, we employ a fully probabilistic diffusion model that explicitly samples uncertainty in initial market size and demand expectations. This is particularly key when deploying logistic diffusion curves that are highly sensitive to initial conditions. Second, our framework endogenously links short-term capacity boosts and demand-pull policies to long-term deployment probabilities, quantifying their relative influence (e.g., the dominant role of early capacity). Third, we make this interaction transparent, whereas earlier studies typically left it implicit. As such, our analysis completements, but also extends beyond, previous qualitative insights^1,22,32^ by attributing quantified contributions to distinct technological analogs and policy levers, while making uncertainty in diffusion trajectories explicit.

Our analysis shows that, if DAC is to make a serious contribution towards achieving the Paris climate targets, policy needs to step in and ensure that growth rates follow the higher end of the analogs spectrum and that short-term DAC capacity is boosted. Especially, boosting short-term capacity may be crucial. Instruments like public investments (e.g., the US Department of Energy 3.1$ billion investments in DAC hubs) and subsidies (e.g., the U.S. 45Q tax credit) can stimulate early deployment and achieve a similar effect to what simulated in our analysis. These policy implications rely, however, on two critical assumptions: (i) that analogies to prior technologies hold, and (ii) that DAC deployment approximately follows logistic growth dynamics. In reality, nascent technologies such as DAC may not follow a logistic growth in the first phases of their deployment^26,34^. While this study sheds light on the challenge that DAC faces if it is to play a role at a climate-relevant scale by mid-century, ultimately, the diffusion of DAC will depend on cost-competitiveness and technological, market, and policy conditions that are hard to predict a priori, and are well beyond the scope of this study. As such, our findings are illustrative rather than prescriptive.

In the absence of serious efforts to multiply the short-term DAC capacity, our findings highlight the risks of over-relying on future large-scale DAC deployment to meet our climate targets, as this could lead to potential carbon lock-in. Policymakers should thus balance efforts to scale DAC with advancing other carbon removal technologies and prioritizing aggressive emissions reductions^35–37^. In the short term, policymakers should encourage innovation and deployment to achieve near-term momentum, while acknowledging that the technology’s current uncertainties may limit the feasible DAC potential by 2050. As a result, policymakers should prepare to complement DAC with other carbon removal and mitigation strategies and iteratively review their expectations for the future role of DAC, based on tracking its deployment and growth trajectory.

## Methods

To explore diffusion pathways for DAC deployment and assess the impact of enhanced policy, we use a stochastic logistic (S-shaped) diffusion framework, which is widely used to describe the adoption of technologies entering finite markets^24,38,39^. Such diffusion pathways are characterized by three phases: a formative phase with slow and uncertain growth, a growth phase marked by rapid expansion driven by learning and cost reductions, and a saturation phase in which deployment slows as market limits are approached. Previous studies have shown that clean energy and carbon dioxide removal (CDR) technologies can be reasonably approximated by such S-shaped trajectories^40–42^, and we adopt this stylized representation for DAC deployment (Supplementary Method 1, Supplementary Fig. 1).

Our modeling framework builds on and expanded the probabilistic diffusion model initially developed by Odenweller et al.^24^, which originally applied to green hydrogen and in more recent studies was applied to sustainable aviation fuels^25^. Rather than producing point forecasts, the model generates probabilistic feasibility spaces of DAC deployment by explicitly sampling uncertainty in key diffusion parameters (Supplementary Method 2). The model is deliberately parsimonious and does not represent techno-economic cost dynamics, investment behavior, or explicit climate policy pathways. Instead, it is designed as a “what-if” experiment to explore plausible DAC deployment trajectories conditional on empirically observed diffusion patterns of analogous technologies.

### Key uncertain parameters

In our modeling framework, DAC diffusion is governed by three independent uncertain parameters (Supplementary Method 3): (i) initial installed capacity, (ii) the emergence growth rate, and (iii) long-term demand pull.

Initial capacity defines the starting point of the diffusion curve and represents the cumulative global DAC capacity expected to be operational by 2030. To estimate the initial capacity, we compiled a database of existing, under-construction, and announced DAC projects by integrating existing sources^3,23,30,43–46^. Uncertainty arises from project attrition, delays, and non-realization of early-stage announcements. We therefore treat initial capacity as a probabilistic input rather than a fixed value.

The emergence growth rate determines the maximum annual growth rate attained once DAC exits its formative phase and enters rapid expansion. Rather than extrapolating DAC growth directly, we parameterize this rate using historical diffusion patterns of three technological analogs: ammonia synthesis (baseline), LNG (pessimistic sensitivity), and wind energy (optimistic sensitivity). These analogs were selected to span a wide range of historically observed growth rates and based on the analysis by Roberts and Nemet^21^, who identified them as suitable analogs on the basis of their scaling potential, high complexity and moderate adaptability as per Malhotra and Schmidt^27^ and Sievert et al.^2^. To estimate the emergence growth rates, we combined existing databases^21,30,32,47,48^ and treat it as stochastic, sampling from empirically derived truncated distributions based on the exponential growth phases of each analog technology (see Supplementary Method 1–6).

To simulate demand for DAC, we defined a steadily increasing demand trajectory that reaches the long-term demand size by 2050. A demand-pull anticipation described the ‘ability’ to anticipate the demand size in 5 years’ time, and therefore grow the DAC capacity to meet that demand (Supplementary Method 4). These demand pulls represented the political, regulatory, and competitiveness-enhancing effects that expand market opportunities, such as the inclusion of DAC in voluntary and compliance carbon markets (e.g., Paris Agreement Article 6 mechanisms, EU Emission Trading Systems) or setting specific DAC targets as part of the nationally determined contributions to the Paris Agreement. To reflect the high uncertainty in long-term DAC demand, the long-term demand size was randomly sampled in each model run from a uniform distribution (Supplementary Fig. 4) ranging between 0 and 5.1 GtCO₂ yr⁻¹ by 2050 for the base case, based on a bottom-up sector-specific assessment of global climate mitigation scenarios. For the maximum potential demand of 5.1 GtCO₂ yr⁻¹ by 2050, we included DAC-based carbon in the hard-to-abate sectors, including aviation, maritime shipping and chemicals, as well as cross-sectoral CDR (Supplementary Tables 4, 5). This approach ensures that our results do not depend on a single assumed benchmark, but instead reflect the wide distribution of possible DAC futures, which in turn are affected by uncertainties such as the future CDR requirements, the future role of net-zero aligned carbon capture and utilization, and the competitiveness of DAC in carbon markets and compared to other CDR methods.

Using the logistic diffusion model (Supplementary Method 5, 6), the initial capacity determined the y-axis intercept, the emergence growth rate influenced the slope, and the demand pulls defined the asymptote of the S-shaped diffusion curve, reflecting the introduction of the CDR market by DAC.

While acknowledging their real-world interdependencies, such as that early project performance can influence both perceived growth potential and long-term demand credibility, we treat these three levers as independent in the model by design. This simplification allows us to isolate and quantify the relative influence of each lever on DAC diffusion outcomes, consistent with the probabilistic feasibility-space approach and with the aim of preserving analytical clarity rather than reproducing every feedback of the real system (see Supplementary Methods 2).

### Policy scenarios

In a subsequent step, we extended this framework to include additional policy levers. This involved forming further probabilistic feasibility spaces under more optimistic conditions then in the base case, including (Supplementary Method 4):

Short-term capacity pushes by 2030: this lever emulates a more aggressive initial capacity expansion, which could be for example the result of government investment and purchasing programmes (such as the DAC hubs from the US Department of Energy^49^ or the potential EU CDR purchasing programme^50^) or of the entrance of new market actors (e.g., Asian countries). In the model, the effect of this lever has been mimicked by increasing the 2030 capacity of DAC by a factor 10. While optimistic, in median this factor 10 increase roughly matches the 2030 DAC capacity projected by modeling and scenario exercises^51,52^.

Long-term demand credibility: this lever emulates a case with better investors’ foresight and regulatory certainty, for example, due to better market conditions leading to safer long-term investments. To mimic the dynamic of this lever, we increase the anticipation of the DAC demand by a factor 3, reflecting a shift from a medium-term to a long-term investment horizon^53^.

Minimum long-term demand: this lever guarantees a minimum DAC demand by 2050 and thus emulates the impact of policies such as 2050 DAC-specific targets, for example in national governments’ long-term climate strategies, or DAC-specific mandates and public provision programmes. In the model, we mimic the effect of this lever by constraining the lower range of 2050 DAC targets to 1.79 GtCO₂ yr⁻¹, which is 35% of the estimated maximum upper boundary of global DAC demand in 2050. This reflects a hypothetical higher level of ambition for DAC (Supplementary Tables 5, 6).

These levers are implemented individually and in combination to assess their relative influence on DAC deployment trajectories.

### Monte Carlo simulation

We generate probabilistic DAC diffusion pathways using Monte Carlo simulation (*N* = 1000 runs per scenario) (Supplementary Methods 5, 6). In each run, values for initial capacity, emergence growth rate, and long-term demand are randomly drawn from their respective distributions and propagated through the adjusted logistic diffusion model. This approach yields distributions of possible DAC deployment trajectories rather than single outcomes, allowing us to assess median pathways, uncertainty ranges, and tail outcomes.

## Supplementary information

Supplementary Information

Transparent Peer Review file

## Data Availability

All data analyzed in the paper have been derived from previously published materials, which are included in the listed references and are publicly available in the GitHub repository and permanently archived on Zenodo at 10.5281/zenodo.18526803. Source data underlying the main figures are provided with this paper. Supplementary data are provided in the Supplementary Information.

## Code availability

All code necessary for replicating reported results is available at GitHub and archived on Zenodo at 10.5281/zenodo.18526803.
