# Supplementary material for: Short-term action is key for gigaton-scale Direct Air Capture by 2050
Source: Nat Commun. 2026 May 9;17:6184. doi: 10.1038/s41467-026-72691-3 (PMC13370031; doi:10.1038/s41467-026-72691-3)
Supplement: Supplementary file 1 — Supplementary Information [file 41467_2026_72691_MOESM1_ESM.pdf]

Supplementary Information

Short-term action is key for gigaton-scale Direct Air Capture by 2050

Tatjana Zurbriggen[1], Nicoletta Brazzola[1,2,3*], Adrian Odenweller[4], Falko Ueckerdt[4,5], Joeri Rogelj[6,7]

1. Institute for Environmental Decisions (IED), ETH Zürich, Zurich, Switzerland
2. Earth Sciences Department, University of Oxford, Oxford, UK
3. German Institute for International and Security Affairs (SWP), Berlin, Germany
4. Potsdam Institute for Climate Impact (PIK), Potsdam, Germany
5. Interdisciplinary Transformation University Austria, Linz, Austria
6. Centre for Environmental Policy (CEP) and Grantham Institute – Climate Change and Environment, Imperial College London, London, UK
7. Energy, Climate and Environment Program, International Institute for Applied Systems Analysis (IIASA), Laxenburg, Austria

* Correspondence to: nicoletta.brazzola@swp-berlin.org

TABLE OF CONTENTS

Supplementary Method 1: Theoretical background 1

Supplementary Fig. 1 **Stochastic S-curve framework for direct air capture (DAC) deployment under policy uncertainty**..1

Supplementary Method 2: Model overview 2

Supplementary Fig. 2 **Modelling outline of DAC deployment analysis**. 2

Supplementary Table 1 **Overview of all relevant databases and references for performing the analysis**..3

Supplementary Table 2. **Overview of different studies and our approach to complement and combine them**...3

Supplementary Method 3: Uncertain Parameters 4

Supplementary Table 3. **Overview of uncertain parameters for the base case and the case with enhanced policy.** 4

Supplementary Fig. 3.1 **Overview of historical, future planned and announced DAC projects**..5

Supplementary Fig. 3.2. **Probability distributions of initial capacity in 2030**……………. ………6

Supplementary Fig. 4. **Distribution of emergence growth rates and global DAC 2050 targets**.. 7

Supplementary Table 4. **Summary Table of the minimum and maximum long-term DAC demand market size.** 8

Supplementary Table 5. **Maximum and minimum DAC demand pull values** 8

Supplementary Method 4: Enhanced policy scenario consisting of three policy levers 9

Supplementary Method 5: Truncated Normal Distribution...10

Supplementary Fig. 5. **Exponential growth phases used for ammonia synthesis in the period 1932 to 1959, LNG in the period 1974 to 2020, and wind energy in the period 1997 to 2023.**11

Supplementary Method 6: Logistic Technology Diffusion Model……………………………………12

Supplementary Table 6. **Overview of uncertain parameters**..13

Supplementary Notes 14

Supplementary Figures 14

Supplementary Fig. 6. **Regional DAC diffusion feasibility space until 2100**. 14

Supplementary Fig. 7. **Probability feasibility space for achieving regional DAC deployment by 2100 globally, based on ammonia synthesis growth rates.**. 15

Supplementary Fig. 8. **Probability feasibility space for achieving regional DAC deployment by 2100 in Europe, based on ammonia synthesis growth rates.**..16

Supplementary Fig. 9. **Probability feasibility space for achieving regional DAC deployment by 2100 in North America, based on ammonia synthesis growth rates**..17

Supplementary References 18

SUPPLEMENTARY INFORMATION

Supplementary Method 1: Theoretical background

We modelled the diffusion of Direct Air Capture (DAC) using an S-shaped curve, which technologies typically follow when entering a finite market [1–3]. The curve, which initially rapidly grows and then flattens as saturation approaches, reflects how early adopters create niche markets, enabling wider adoption as conditions improve, such as better performance or lower costs. Green, clean and CDR niche technologies can also be approximated by such an S-shaped curve to predict a so-called technology diffusion pathway [4–6]. Consequently, our analysis also assumed this approximation for DAC deployment.

The diffusion pathway progresses through three phases: formative, growth, and saturation. The formative phase involves gradual, uncertain growth as demonstration projects face technical challenges and high costs, often described as the "valley of death" [7]. The growth phase sees accelerated market adoption driven by cost-reducing learning effects [8,9]. Finally, the saturation phase occurs when growth slows due to techno-economic and social factors, reaching the market's final level [3]. Such paths, which are modelled with the help of a logistical function, captures the full technology adoption process accordingly (Supplementary Fig. 1).

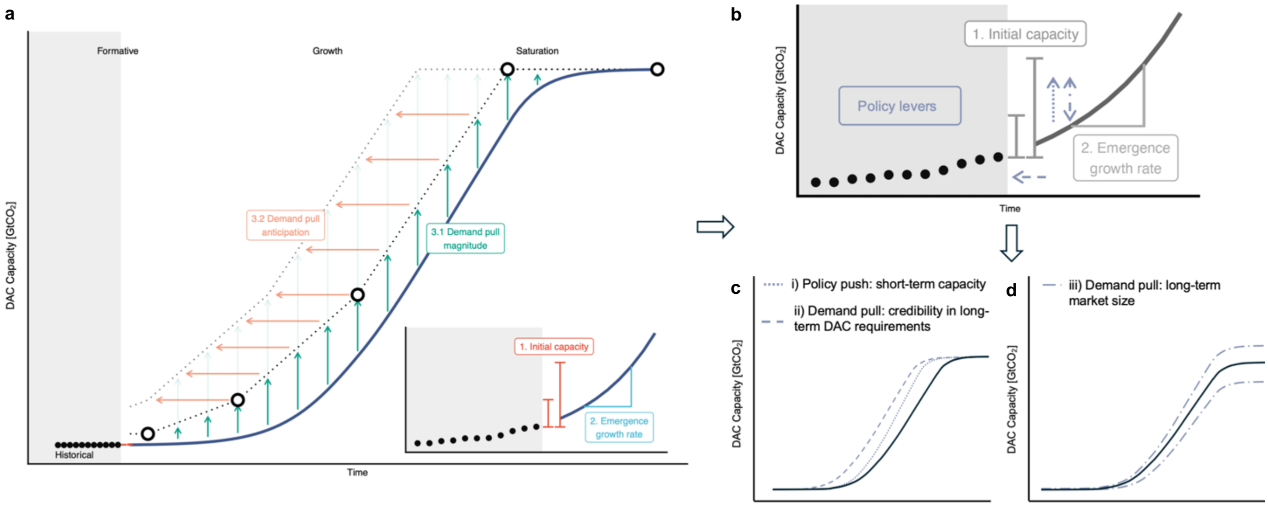

Supplementary Fig. 1 **Stochastic S-curve framework for direct air capture (DAC) deployment under policy uncertainty.** (**a**) Conceptual illustration of the modified logistic (S-curve) diffusion model following Odenweller et al.[1], extended to represent uncertainty and policy levers affecting Direct Air Capture (DAC) capacity deployment. Uncertain parameters include the initial installed DAC capacity (parameter one), the intrinsic growth rate (parameter two), and demand-pull dynamics (parameter three). Historical global DAC capacity is shown as black filled circles with a shaded uncertainty envelope. Larger black open circles indicate deployment projections from Odenweller et al.[1], which define the reference magnitude of demand pull (sub-parameter three point one). A grey dotted line represents the demand pull defined by linear interpolation between these deployment projections. The magnitude of demand pull is illustrated by green arrows and is modified in this study through minimum long-term demand security (policy lever three). Anticipation effects and time-varying demand pull are illustrated by orange arrows and are parameterized using long-term demand credibility (policy lever two). (**b**) Close-up of the formative phase highlighting uncertainty in initial DAC capacity. Uncertainty is represented using simplified error bars, where the minimum corresponds to the cumulative capacity of projects currently operating or under construction, and the maximum includes all announced projects irrespective of development certainty. Grey-blue lines illustrate the effects of policy levers on example diffusion paths: (**c**) short-term capacity boosts until 2030 (policy lever one) steepen early growth; long-term demand credibility (policy lever two) shifts diffusion forward in time via anticipation effects; (**d**) and minimum long-term demand security (policy lever three) constrains long-term market size and generates stochastic deployment outcomes. The solid S-shaped curve represents a single illustrative diffusion path within the resulting feasibility space.

Supplementary Method 2: Model overview

Supplementary Fig. 2 conceptually illustrates the full modelling workflow adopted in this study, which simulates the probabilistic diffusion pathway for DAC deployment, depending on the influence of policy levers simulated using a logistic function. We defined the function by three uncertain and independent main parameters: initial capacity, emergence growth rate and demand pull. In our modelling framework, these parameters are treated as independent. This is a simplifying assumption: in reality, early project success or failure, socio-environmental challenges, and cost dynamics can create feedback between these factors. For example, if many early projects were to fail, this could undermine investor confidence and slow down both growth rates and long-term demand pull, as has recently been observed in the case of green hydrogen. Nevertheless, we chose independence for three reasons: (i) to isolate the effect of each factor and assess which has the largest impact on DAC diffusion, (ii) because historical evidence suggests that many technologies still follow logistic (S-curve) pathways even in the presence of such interdependencies, and (iii) because in our implementation, the emergence growth rate starts only at the defined initial capacity, such that initial capacity sets the starting point while the slope and asymptote are determined separately.

To estimate the initial capacity and emergence growth rates for analog technologies, we relied on the combination of existing databases and determined these parameters by applying truncated normal distributions (Supplementary Table 2). Further, for the emergence growth rate parameters we used the same technology analogs regarding the base case and the enhanced policy case. We parameterized the emergence growth rates using the distribution of exponential growth rates (Supplementary Fig. 3, Supplementary Fig. 4) of ammonia synthesis, liquefied natural gas (LNG) and wind energy, whereas the key focus was on ammonia synthesis as a baseline and the latter two technologies functioned as optimistic and pessimistic growth rate sensitivities (Table 2). An overview of the data used can be found in Supplementary Table 1.

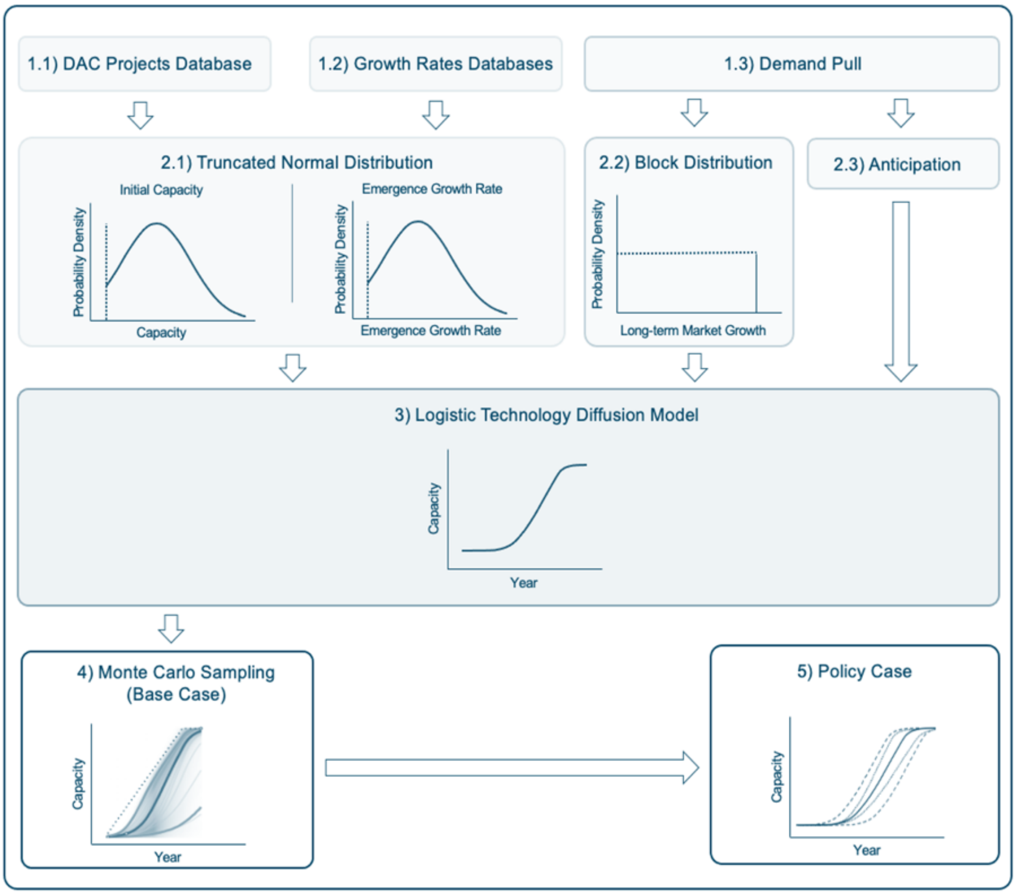

Supplementary Fig. 2 **Modelling outline of DAC deployment analysis.** Definition of uncertain parameters (initial capacity, growth rate, demand pull), creation of probabilistic feasibility spaces using an adjusted logistic diffusion model as well as Monte Carlo sampling (base case) and subsequent adjustment regarding different policy levers to analyse their influence (case with enhanced policy).

Supplementary Table 1 **Overview of all relevant databases and references for performing the analysis.**

Type of Information	Description	References
DAC projects database	Created database of all historical and future announced DAC projects (Supplementary Methods 3)	[10–17] 'Capacity DAC Projects Database 2024.xlsx' in the accompanying GitHub repository and on Zenodo[18].
Growth rate of ammonia synthesis and pessimistic growth rate sensitivity (LNG)	Growth rates based on most suitable analog technology to DAC (Supplementary Methods 3)	[19]
Optimistic growth rate sensitivity wind energy	Growth rates based on market-driven capacity data for wind energy (Supplementary Methods 3)	[20]
Long-term market size	Estimation of market size range for DAC in 2050 based on climate mitigation scenarios (Supplementary Methods 3)	[12,21,22]
Code Availability and Additional Excel Files	Provided Code and Excel files for the mentioned databases and calculations	GitHub, Zenodo[18]

Our modeling framework is largely based on the probabilistic diffusion model for green hydrogen, developed by Odenweller et al.[1]. This model integrates two approaches: modifying growth models with historical asymptotic parameters[23,24] and using historical development to set ex-ante targets for wind and solar energy[25]. Odenweller et al.[1] synthesized these approaches to create probabilistic feasibility spaces for green hydrogen diffusion, which parallels the current stage of DAC technology. Therefore, their model built the basis for our further developed and optimized analysis.

For the growth rates chosen in our analysis (ammonia synthesis, LNG and wind energy), we relied on the study of Roberts and Nemet[19]. This is because on the one hand, they developed specific criteria to identify suitable technological analogs, focusing on technologies with at least 20 years of history, significant scaling potential, and high complexity and moderate adaptability. On the other hand, they used the findings of Malhotra and Schmidt[26], which emphasized the role of complexity and customization in technology adoption, to assess the suitability of different technologies. Lastly, the analysis of Sievert et al.[27] confirmed the high complexity and moderate customization of liquid DAC, which further supported the selection of relevant technological analogs and provided a solid basis for the analysis of scaling potential.

Supplementary Table 2 gives an overview of the mentioned and additionally relevant studies and how we used them for our approach and therefore how it differs from other literature.

Supplementary Table 2. **Overview of different studies and our approach to complement and combine them.**

Available Studies	Usage for our approach	Differences to our approach
Odenweller et al.[1]	Adapted and elaborated logistic model.	Focus on green hydrogen with distinction of conventional (wind energy and solar PV) and unconventional (fast growing historical

		technologies) growth rates and a fixed demand pull for the hydrogen market in 2050.
Roberts and Nemet[19]	Identifiction and database of analog technologies for liquid DAC (ammonia synthesis, carbon capture & storage (CCS) and LNG).	Instead of additionally using LNG and CCS as analog technology, we added them only as sensitivities and replaced the analog CCS with wind energy, firstly to include solid DAC and secondly to have an optimistic growth rate scenario for comparison. The analog LNG served accordingly as a pessimistic growth rate scenario.
Edwards et al. [28]	Recognition of their approach.	Our model includes extended probabilistic elements for realistic market development and a more systematic choice of analogs. Methodological approach based on Integrated Assessment Models (IAMs).
Nemet et al.[17]	Growth rate data of LNG from HATCH database.	Methodological approach based on IAMs, focusing mainly on DACCS and don't model policy uncertainty in their framework.

Supplementary Method 3: Uncertain Parameters

In the base case, the uncertain parameters influencing DAC's development are defined by the initial capacity in 2030, the emergence growth rate, and the long-term market size. These parameters are informed by a combination of existing data, expert judgment, and historical analogs and are summarized in Supplementary Table 3.

Supplementary Table 3. **Overview of uncertain parameters for the base case and the case with enhanced policy.** Details regard the baseline emergence growth rate of ammonia synthesis (see Supplementary Table for wind energy and LNG).

<table>
<tr><td rowspan="5"></td><td colspan="11">Uncertain parameters</td></tr>
<tr><td colspan="7"></td><td colspan="4">Uncertain levers</td></tr>
<tr><td colspan="3">Initial capacity (2030)</td><td colspan="4">Emergence growth rate (Ammonia Synthesis)</td><td>Short-term capacity factor</td><td>Demand anticipation</td><td colspan="2">2050 demand size</td></tr>
<tr><td>Min</td><td>Mean</td><td>σ</td><td>Min</td><td>Mean</td><td>Max</td><td>σ</td><td colspan="2"></td><td>Min. Value</td><td>Max. Value</td></tr>
<tr><td colspan="3">[$MtCO_2\ yr^{-1}$]</td><td colspan="4">[% yr^{-1}]</td><td>[the factor by which the 2030 initial capacity in the base case is increased]</td><td>[years]</td><td colspan="2">[$GtCO_2\ yr^{-1}$]</td></tr>
<tr><td>Base Case</td><td>4.8</td><td>8.8</td><td>0.95</td><td rowspan="2">0.0</td><td rowspan="2">11.0</td><td rowspan="2">25.5</td><td rowspan="2">5.0</td><td>1.0</td><td>5.0</td><td>0.0</td><td>5.1</td></tr>
<tr><td>Case with enhanced policy</td><td>48.0</td><td>88.0</td><td>9.5</td><td>10.0</td><td>15.0</td><td>1.79</td><td>5.1</td></tr>
</table>

<u>Initial capacity in 2030</u>

The momentum driving DAC's removal capacity from the formative to the growth phase is uncertain, but potentially significant in the coming years. Since the initial capacity depends on this momentum, we treated it as an uncertain parameter in our analysis. We compiled data for the initial capacity analysis from the International Energy Agency (IEA) Carbon Capture, Utilisation, and Storage (CCUS) Project Database[13], the State of CDR Report[12,29] and other individual sources (see also 'Capacity DAC Projects Database 2024.xlsx' in the accompanying GitHub repository[54]). Using these, we created a comprehensive database of historical and future DAC projects, detailing their locations, development statuses, technological characteristics, and removal capacities. We reclassified projects labeled "Proof of Technology" as "Operational" or "Decommissioned" depending on whether they are still in operation, resulting in a database of 78 entries. This data was then used to compute the initial capacity of existing, planned, and announced projects and to parameterize the distribution of cumulative initial capacity based on project status (Supplementary Fig. 3.1). The mean of the normal distribution of the initial capacity is 8.8 $MtCO_2\ yr^{-1}$, while the standard deviation is 0.95 $MtCO_2\ yr^{-1}$. To account for the fact that some plants are already operational or currently being built, and thus we can rely on their capacity by 2030, we truncated the distribution on the lower end, corresponding to 4.8 $MtCO_2\ yr^{-1}$ by 2030 (Supplementary Fig. 3.2).

We selected 2030 as the initial year, assuming that it takes around five years to initiate the necessary political measures for the realization of the projects and thus to implement the corresponding projects from announcement to commissioning. This assumption accounts for the uncertainty of project implementation before a Final Investment Decision ("FID") and recognizes that even projects with a "FID" may face delays. Therefore, this approach balances short-term dynamics with the exclusion of uncertain long-term announcements. These considerations apply to the base case in our analysis, which excludes the application of targeted policy levers.

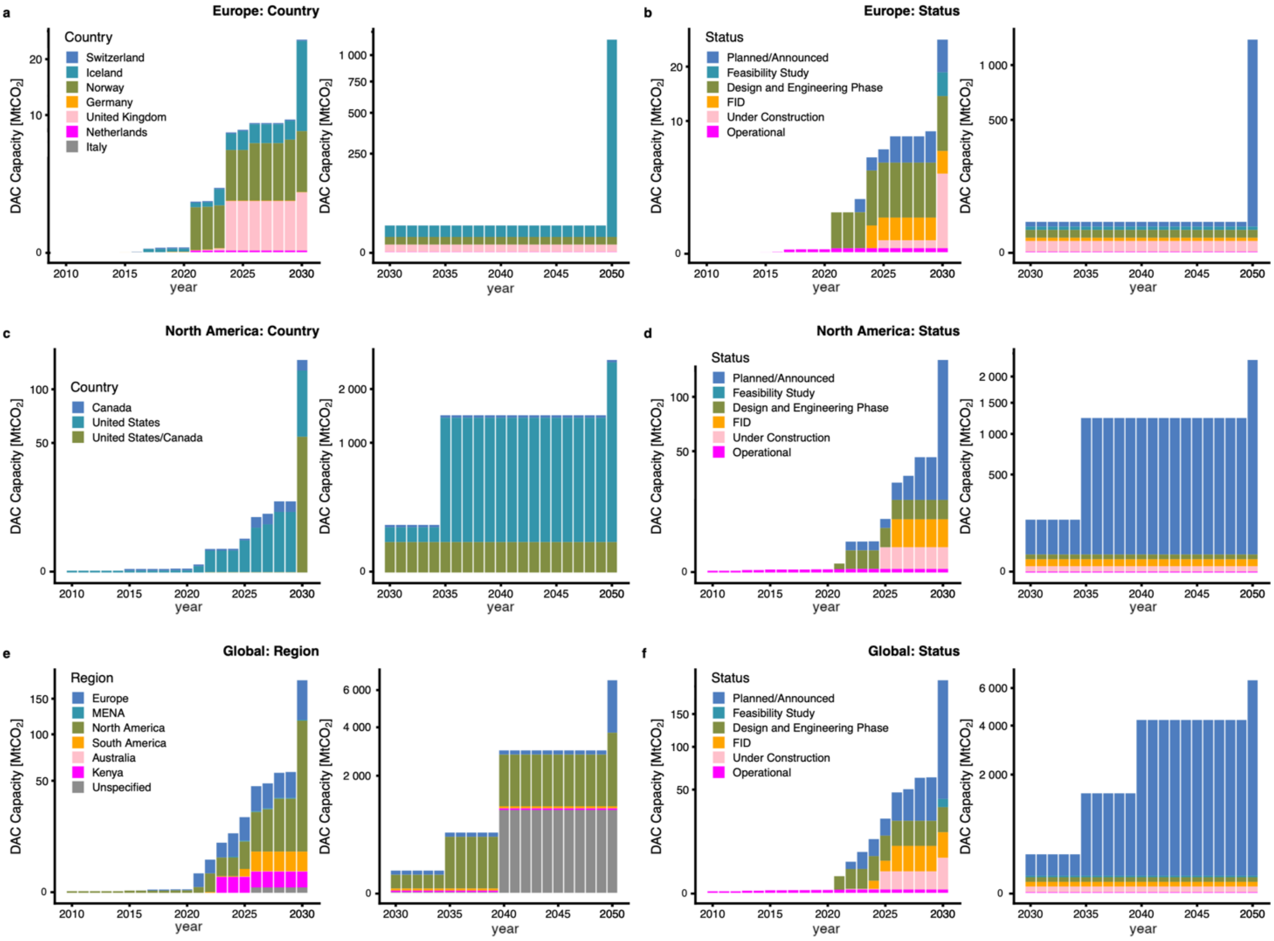

Supplementary Fig. 3.1 **Overview of historical, future planned and announced DAC projects. a-d**, show projects of the regions Europe (**a**) and North America (**c**) based on the countries and their project development status (**b, d**). Furthermore, (**e-f**) show a global project overview based on regions (**e**) and their development status (**f**). Due to the discrepancy in project capacities, the plots were split between 2010 to 2030 and 2030 to 2050 and

compressed on the y-axis. The decommissioned projects were not included in the figure due to their marginal quantity.

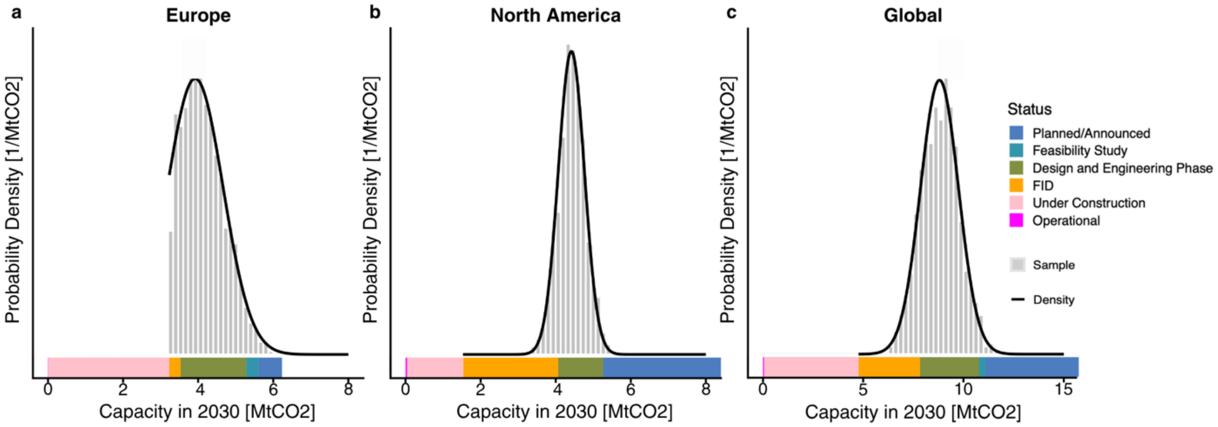

Supplementary Fig. 3.2. **Probability distributions of initial capacity in 2030.** Panels show the initial capacity in Europe (**a**), North America (**b**) and on a Global level (**c**). In addition, the horizontal bars represent the capacity in 2030 based on Figure 3.1 and without decommissioned projects. Given this distribution, the mean value for the initial capacity in 2030 results in 3.92 $MtCO_2$ for Europe, 4.42 $MtCO_2$ for North America and 8.8 $MtCO_2$ for the Global capacity.

Emergence growth rate

Possible cost reductions, political and government support, and technological maturity influence the growth rate of new CDR technologies [11,12,30,31]. Given these uncertainties, we included the growth rate of DAC as an uncertain parameter in our analysis. In this study, we focused on the maximum annual growth rate, known as the emergence growth rate [32], rather than the gradually declining rate due to market saturation. The emergence growth rate, realized after the formative phase, was parameterized using the slope parameter in the logistic function, following Odenweller et al. [1].

To determine the different emergence growth rates, we used empirical data from the most suitable technology analogs for liquid and solid DAC, with having the goal to obtain different growth rates for DAC and therefore a range of possible deployment scenarios, defining a main baseline case and a pessimistic and an optimistic case as sensitivities for the analysis:

Ammonia Synthesis and pessimistic LNG scenario: For the liquid DAC technology analogs, we followed the methodology of Roberts and Nemet[19]. With their approach we identified ammonia synthesis and LNG for technology analogs of liquid DAC in our analysis. We focused on ammonia synthesis for our baseline scenario, since this analog is the most suitable analog for liquid DAC. For our pessimistic scenario we chose LNG, since this technology showed a similar but more pessimistic growth then ammonia synthesis[19].

Optimistic wind energy scenario: Since our methodology expand on solid DAC, we also included an optimistic scenario based on the emergence growth rate of wind power as a third analog technology. We based the choice of wind energy as a third analog technology for solid DAC based on the complexity and modularity per Malhotra and Schmidt [26] and per Sievert et al.[27].

Instead of comparing the same technology across different regions, we considered different technologies within the same region, as historical growth rates for DAC and similar technologies, like green hydrogen, primarily reflect past political support rather than future potential[33]. This approach also simplified scenario construction by not requiring consideration of all factors influencing technology diffusion speed [1]. Lastly, since the uncertainties of these emergence growth rate parameters were also unstable and non-linear, as in Odenweller et al. [1], we expressed this scattering using a Monte Carlo simulation approach in the logistic diffusion model.

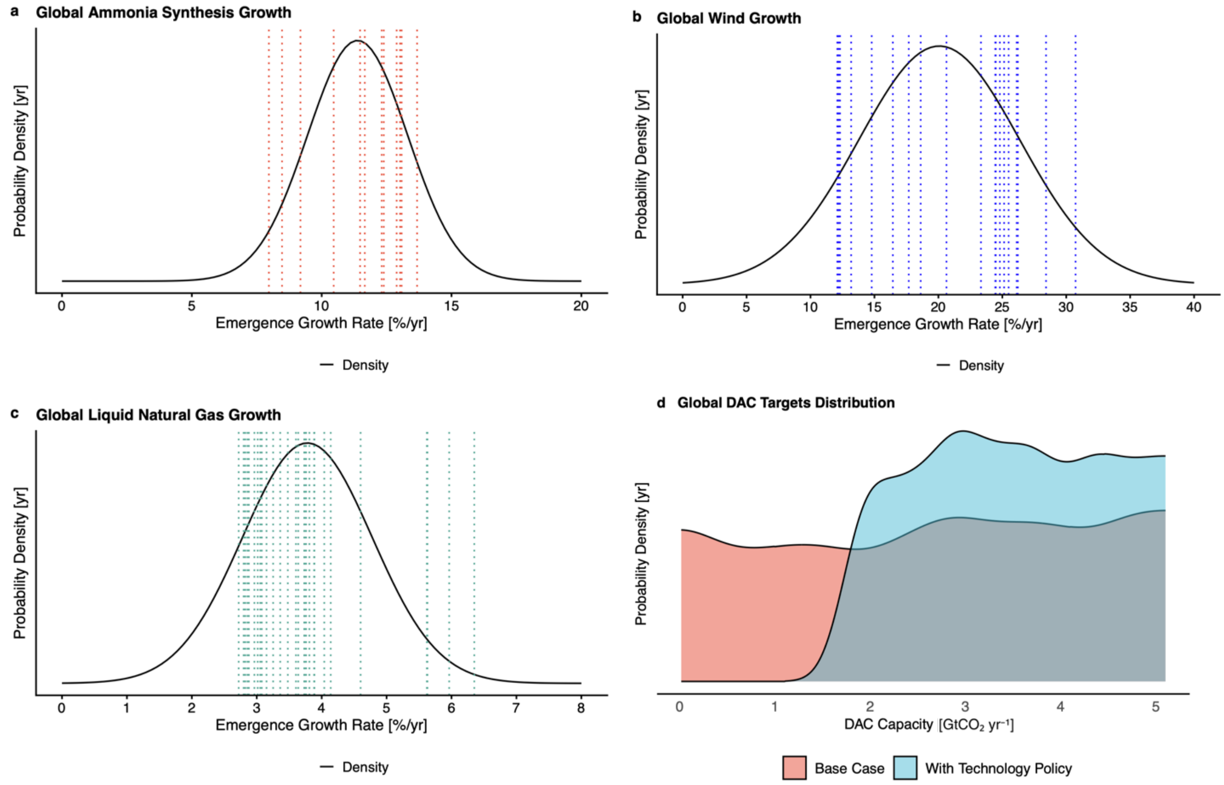

Supplementary Fig.4. **Distribution of emergence growth rates and global DAC 2050 targets.** ***a-c***: Probability distributions of the analysed global emergence growth rates, ammonia synthesis (***a***), and wind (***b***) as well as LNG (***c***) for sensitivity scenarios. The vertical dotted lines in (***a-c***) represents the 7-year moving intervals, which were determined through fitted exponential models to the historical data of the analogs and with which the emergence growth rate distribution of the corresponding technologies was parametrised. (***d***) Uniform distribution of the respective minimum and maximum values for the long-term market demand pull of the DAC capacity in 2050, with the distributions for the base case shown in red and the one with enhanced policy in blue.

Demand pull and long-term market size

As the outcome of competition between different climate mitigation measures has not yet been determined for many end applications, the final CDR market volume is characterised by uncertainty [1,34]. In these applications, DAC is not only a new CDR technology in itself, but its supply, demand and infrastructure must be developed simultaneously [11,14,30,31,35]. This stands in contrast to, for example, solar and wind energy, which are already fully embedded in the electricity market and its infrastructure. Therefore, the demand pull represented political, regulatory and competitiveness-enhancing effects that increase market opportunities. Due to the uncertainties mentioned above, we included the demand pull, which was differentiated in long-term market size and in credibility in long-term DAC requirements, as a third uncertainty parameter in the analysis.

Long-term market size: In contrast to Odenweller et al [1], we did not use a fixed market size until 2050 for DAC. Instead, we employed a Monte Carlo approach, drawing from a uniform distribution of possible long-term DAC market size by 2050, to reflect the unpredictability of future demand and market dynamics for DAC. Future demand for DAC is highly uncertain, as it depends on the combination of DAC integration in markets (e.g., compliance carbon markets, synthetic hydrocarbon markets, etc.) and on the degree of DAC-specific policy support to enhance its competitiveness relative to lower-cost alternatives (e.g., nature-based CDR, biofuels, etc.).

The range of uncertain DAC demand by 2050 is based on a sector-specific assessment of global climate mitigation scenarios (Supplementary Table 5). We take a bottom-up approach and assess the maximum contribution of DAC in each sector rather than relying on model estimates of future DAC demand, which diverge strongly due to varying assumptions. In the base case, we therefore assumed a range between a minimum demand of 0 $GtCO_2\ yr^{-1}$ and a maximum demand of 5.1 $GtCO_2\ yr^{-1}$. The lower bound represents a scenario where DAC fails to scale or is outcompeted by alternative CO_2 sources or removal technologies. The upper bound reflects an extreme yet informative case, constructed “bottom up” from potential DAC demand in both (i) carbon dioxide removal (DACCS) and (ii) carbon utilization (DACCU) applications.

There is ongoing debate regarding whether DAC-based carbon utilization (DACCU) should be included in future DAC demand estimates. Techno-economic assessments[36–39] highlight that DACCU is likely to remain an expensive and uncertain pathway for several decades, given its dependence on low-emission hydrogen. An exception may be the aviation sector, where DAC-based fuels may be cheaper than offsetting strategies based on DACCS under a climate neutrality goal[37]. However, there is current political momentum that hints to the potential role of DACCU in some hard-to-abate sectors, such as aviation. Initiatives such as ReFuelEU Aviation[40] and national mandates for renewable fuels of non-biological origin (RFNBOs) create a regulatory pull for DAC-derived CO_2 feedstocks, which is currently unmatched by similar policies specifically pulling demand for DACCS, as this technology tends to compete with cheaper nature-based and biomass-based solutions under current policy frameworks and carbon markets[12,41,42]. Finally, DACCU is often found to be a more acceptable mitigation solution than DACCS by the public due to its non-reliance on geological storage and due to the fact that the risk of delaying emission reductions is avoided[43–45]. These emerging political and social drivers could stimulate DACCU deployment in the short-ter. In these contexts, DACCU may serve as an early pathway that accelerates DAC learning and infrastructure build-out in the near term[45].

To identify an optimistic, yet plausible upper end of DAC demand by 2050, we proceeded as follows. For the DACCS component, we assume a 50% contribution of DAC to novel CDR needs consistent with the 75th percentile of scenarios limiting warming to below 2 °C by 2050[12]. This assumption is based on the rational that lower cost durable CDR options may have a constrained potential[46]. For the DACCU component, we included potential DAC-based CO_2 use in hard-to-abate sectors such as aviation, maritime transport, and chemical production, where non-biomass-based synthetic fuels and feedstocks may be required to meet future energy and climate targets[47]. We assumed a carbon intensity of fuels and feedstocks of 250 g CO_2 kWh^{-1}. Here, too, we maximally assumed that CO_2 from DAC would contribute 50% of the net-zero CO_2 feedstock by 2050. The combination of DACCS and DACCU demand under this high-deployment scenario results in a total potential DAC market size of 5.1 $GtCO_2$ yr^{-1} by 2050, which defines the upper limit of the uniform distribution used in our simulations. While this 50% assumption represents an optimistic boundary—given the current techno-economic challenges of DACCS and DACCU—it captures the potential scale of DAC deployment under policy and market conditions that strongly favor air-captured CO_2 (e.g., inclusion of only durable CDR in the EU Emissions Trading System (EU ETS), mandates such as ReFuelEU Aviation).

Supplementary Table 4. **Summary Table of the minimum and maximum long-term DAC demand market size.**

Case	Minimum Value [$GtCO_2$ yr^{-1}]	Maximum Value [$GtCO_2$ yr^{-1}]
Base case	0	5.1
Case with enhanced policy	1.79	

Credibility in long-term DAC requirements: The demand pull regarding the credibility in long-term DAC requirements reflected investors' foresight and regulatory certainty, indicating their expectations for the duration of demand pull long-term market size projections and the competitiveness of CDR by DAC. For the base case we set the default assumption of this anticipation to five years. This assumption is based on Odenweller et al. [1] and data regarding the market expansion duration of other clean technologies [30,48] (Supplementary Table 5).

Supplementary Table 5. **Maximum and minimum DAC demand pull values**

Sector or application with potential DAC requirement	2050 energy or carbon demand	Total carbon required	Maximum carbon required from DAC	Assumption on deriving the sector-specific DAC requirement from the total annual demands	Source
Aviation	15.27 EJ yr^{-1}	1.1 $GtCO_2$ yr^{-1}	0.5 $GtCO_2$ yr^{-1}	50% of carbon requirements met by DAC	IEA NZE 2050[47]
Maritime	9.9 EJ yr^{-1}	0.7 $GtCO_2$ yr^{-1}	0.3 $GtCO_2$ yr^{-1}	50% of carbon requirements met by DAC (this assumes large role of methanol	IEA NZE 2050[6]

				compared to the carbon-free fuel ammonia)	
Chemicals carbonaceous feedstocks	55 EJ yr^{-1}	3.8 $GtCO_2$ yr^{-1}	1.9 $GtCO_2$ yr^{-1}	50% of carbon requirements met by DAC (this maximum DAC scenario implicitly assumes little waste incineration Carbon Capture and Storage (CCS), less circularity such as mechanical/chemical recycling routes and less bioplastics)	Fritzeen et al. 2023[22] (GCAM), which shows 55-60 EJ yr^{-1} only for chemical feedstocks (in 3 of 4 scenarios). In comparison, the IEA NZE[6] only has 28 EJ feedstock demand in 2050 (due to demand reductions in e.g. plastics compared to a baseline)
Carbon dioxide removal (CDR)	4.6 $GtCO_2$ yr^{-1}	4.6 $GtCO_2$ yr^{-1}	2.3 $GtCO_2$ yr^{-1}	50% of carbon requirements met by DAC (assuming limits in the availability of biogenic carbon for Bioenergy with Carbon Capture and Storage (BECCS), which dominates novel CDR in many IAM scenarios)	Smith et al. 2023 (table 8.2)[29], 75th percentile from novel CDR (e.g. DACCS, BECCS) in C1-C3 scenarios
Sum across sectors		10.2 $GtCO_2$ yr^{-1}	5.1 $GtCO_2$ yr^{-1}		

Supplementary Method 4: Enhanced policy scenario consisting of three policy levers

Since the scale-up and therefore the extent of the deployment of DAC is highly dependent on the political measures taken in the short and long term, it is critical to capture the effects of possible policy levers. This provides policymakers with a guideline on how and to what extent measures must be taken to align with global climate targets. Possible future policy levers that directly influence the diffusion pathway and therefore the deployment of DAC include the influence on the starting capacity in 2030, as well as the impact on the demand pull. The latter can be characterised on the one hand by a growing market size in the long term and on the other hand by anticipation through the creation of credibility regarding long-term DAC requirements. By varying some of the uncertain parameters in Supplementary Methods 3, we define and investigate the impact of three policy levers.

Policy push fostering short-term DAC capacity (in 2030): The initial short-term capacity has a direct effect on the path of the diffusion curve and consequently on the long-term DAC capacity available in 2050. Therefore, by increasing this capacity by a certain factor, more capacity will be available in 2030, which results in a faster scale-up, a quicker emergence of the technology from its niche and ultimately a more probable saturation. For the case with enhanced policy, we therefore assumed the effect of a policy push on short-term capacity by 2030 to be 10 times the initial capacity. This assumption implies that such an increase in capacity is within the realms of possibility, provided that many projects that are currently in "FID" or "Under Construction" project development status transition to "Operational" status by 2030 [48].

Creating credibility in long-term DAC requirements: For the case with enhanced policy, we scaled the default anticipation up to 15 years, based on the assumption that ambitious policy measures have been

taken place. This would make market conditions more stable and safer and allow for a longer foresight horizon.

Securing minimum long-term DAC demand: In the case with enhanced policy, we assumed a more optimistic range of DAC long-term market growth, where political interventions and measures promote more favorable market conditions and therefore higher demand for DAC. The range chosen was restricted to a minimum demand of 1.79 $GtCO_2$ yr^{-1} and the same maximum demand of 5.1 $GtCO_2$ yr^{-1} as for the base case. For the minimum potential demand for DAC-based we assumed 35% of the potential maximum demand of 5.1 $GtCO_2$ yr^{-1}, resulting in a value of 1.79 $GtCO_2$ yr^{-1}. The 35% reflects a general assumption that, by 2050, demand in each hard-to-abate sector will increase by at least this amount, inspired by the projected 35% rise in synthetic aviation fuel (SAF) demand across all EU airports by 2050 [40]. For simplicity, we assumed that the minimum demand for CDR for cross-sectoral carbon capture covers only 17.5% of the total carbon required by 2050, rather than 50% as in the maximum potential demand. This also corresponds to a 35% increase in CDR demand compared to DAC's maximum carbon demand by mid-century.

Supplementary Method 5: Truncated Normal Distribution

The implementation of the stochastic uncertainty analysis was based on the Monte Carlo simulation approach. The parametric uncertainty underlying this approach was reflected by randomly selected samples extracted from probability distributions. For the initial capacity in 2030, as well as for the different emergence growth rates, we applied a normal distribution with a lower truncation.

The lower truncation constrained the distribution to a certain lower bound[49]. We defined this lower bound "a" for the initial capacity distribution by all projects that were already "Operational" or "Under construction" and will start production in 2030. By defining the lower truncation, we were able to determine the truncation interval [a,]. In the next step, we set up suitable conditions for the remaining two degrees of freedom using the µ (mean) and s (standard deviation).

For the first condition (supplementary equation (1)), we assumed that the success rate of the projects with the development status "Feasibility Study" (FS) and "Design and Engineering Phase" (DEP) is 30% due to techno-economic and financial influences [50–52]. Therefore, we set the corresponding post-truncation expected value to this assumed capacity, which can be described as $C_{0.3\ (FSandDEP)}$. Since the probability density function can be expressed as ϕ and the cumulative density function of the normal distribution as Φ, the first condition relates to the expected value (E(X)):

$$E(X) = \mu + \sigma \frac{\phi(\frac{a-\mu}{\sigma})}{1-\Phi(\frac{a-\mu}{\sigma})} = C_{0.3(FSandDEP)} \quad (1)$$

The second condition we determined by assuming a further techno-economic and financial scenario, namely the probability of those projects that have already been confirmed by a "FID" and are therefore actually built is 15%. We labelled this capacity C_{FID}. Using the truncated cumulative distribution function, this condition can be described as supplementary equation (2):

$$F(C_{FID}) = P(X \leq C_{FID}) = \frac{\Phi\left(\frac{C_{FID}-\mu}{\sigma}\right) - \Phi(\frac{a-\mu}{\sigma})}{1-\Phi(\frac{a-\mu}{\sigma})} = 0.15 \quad (2)$$

Due to the scarcity of empirical data to determine these conditions regarding DAC, we adopted the two conditions 2.4.1 and 2.4.2 almost identically to those of Odenweller et al. [1]. However, for the capacity $C_{0.3\ (FSandDEP)}$ in the first condition, we orientated us not only on the success rate of 30% from the source of Odenweller et al. [1] in the case of hydrogen projects, but also on Abdulla et al. [50], which has shown a failure rate of 80% for CCS investments in the US, and on Kazlou et al. [51], which has predicted a failure rate of 76% for today's CCS plans. Furthermore, since we additionally included the DEP projects between the development stages of the FS and the "FID" and aimed for an optimistic and representative distribution, both capacity assumptions of 30% and 15% already made by Odenweller et al. [1] also proved to be valid for our analysis.

To obtain µ and σ, we numerically solved the non-linear system formed by the conditions 2.4.1 and 2.4.2, which allowed us to completely determine the truncated distribution by adding the previously determined truncation value "a". The changed initial capacity of the case with enhanced policy also

passed through this truncated normal distribution, except that that capacity was multiplied by a factor of 10 (Supplementary Methods 4).

For the distribution of baseline growth rates of the analog ammonia synthesis, we extracted the global ammonia synthesis production data from the dataset of Roberts and Nemet (2024)[19]. From this dataset, which contains data between 1924 and 2018, we used only the data from the period 1932 to 1959 to isolate exclusively the exponential growth phase of this technology. We obtained the data for wind energy, which reflected the optimistic sensitivity of the growth rates for DAC, by using the installed wind capacity from the BP Statistical Review of World Energy 2024 [20]. This data was available and therefore used to examine the exponential growth phase of wind energy from 1997 to 2023 for Europe, North America and globally. Our database for LNG capacity, which represented the pessimistic sensitivity of growth rates for DAC, originated from the HATCH database [53], where we looked at a selected period from 1974 to 2020 to capture its exponential growth phase.

We fitted exponential models to the data of each technology in 7-year moving intervals by calculating the mean and standard deviation of the 7-year growth rates of them (Supplementary Fig. 5). We then subsequently used the intervals to parameterize the emergence growth rate distributions of each technology. For the respective distributions, and therefore the different growth rate cases, we assumed a lower truncation of 0% yr^{-1} for all the considered technologies, which we also defined as the lower limit of CDR market growth for DAC. By doing so, we accounted for the possible reality in which the growth of the CDR market for DAC and therefore its adoption has a non-ambitious outcome in the future. We then used the values from the truncated normal distributions for the respective initial capacities of the base case and the case with enhanced policy, as well as for the emergence growth rates, to further calculate the logistic diffusion model and finally to determine the resulting feasibility spaces.

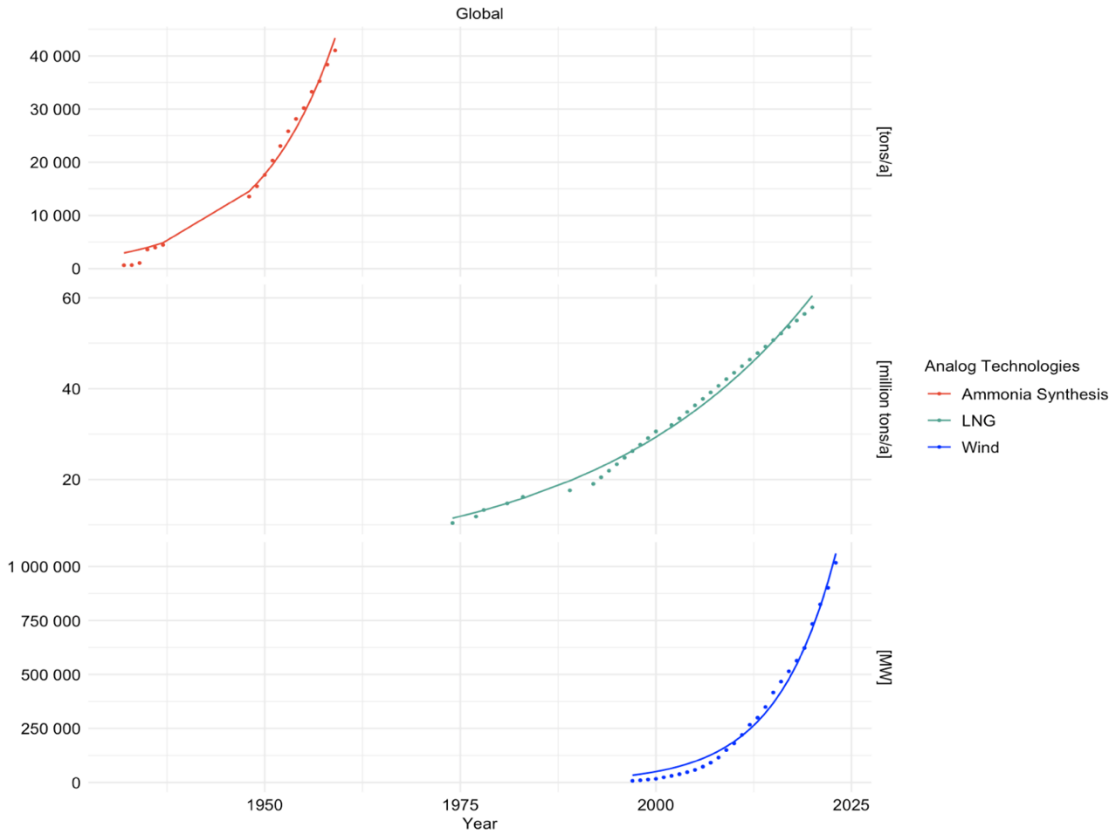

Supplementary Fig. 5. **Exponential growth phases used for ammonia synthesis in the period 1932 to 1959 (pink), LNG in the period 1974 to 2020 (brown) and wind energy in the period 1997 to 2023 (blue**). The exponential models were fitted to the data for each technology at 7-year moving intervals by calculating the mean and standard deviation of the 7-year growth rates for each technology.

Supplementary Method 6: Logistic Technology Diffusion Model

As already introduced in Supplementary Methods 1, we elaborated the adapted logistic technology diffusion model provided by Odenweller et al. [1]. As with the scale-up of green hydrogen in the case of DAC deployment, the demand pull of the long-term market size must also simultaneously expand and harmonise the three definition areas of infrastructure, supply and demand. To realise this, we implemented the standard logistical technology diffusion model in such a way that a steadily growing demand pull was embedded, similar to Odenweller et al. [1] . In contrast to the referenced study, our demand pull approach did not assume a fixed end-market volume. Instead, we considered a range of potential end-market volumes and corresponding market growth trajectories, applying a randomized distribution with the following characteristics (supplementary equation (3)):

$$f(x) = \begin{cases} \frac{1}{(g-c)}, & \text{for } c \leq x \leq g \\ 0, & \text{otherwise} \end{cases} \tag{3}$$

Whereas "c" is the minimum potential demand and "g" the maximum potential demand that can occur in the distribution (Supplementary Methods 3, Demand pull). Furthermore, all values between "c" and "g" have equal probability and the area under the probability density function is equal to 1, as it represents a probability distribution. This method enabled us to determine a randomly selected long-term DAC demand for each Monte Carlo iteration, resulting in varied end-market size outcomes. This range of possible end-market size scenarios we then defined differently between the base case and the case with enhanced policy, based on specific assumptions (Supplementary Methods 3 and 4).

Additionally, since the CO_2 removal by DAC, like green hydrogen, cannot be described representatively by substituting technology shares, as Odenweller et al. [1], we modelled the directly growing market volume, expressed by the CDR capacity of DAC in our analysis, instead of its market shares. In this way, the implementation of the diffusion could be reconstructed as described below:

The standard logistic function for the DAC removal capacity C(t) is described per definition by the asymptote C_{max}, growth constant k, inflection point t_{inf} and Euler's number e = 2.718 (supplementary equation (4)):

$$C(t) = \frac{C_{max}}{1 + e^{-k\,(t-t_{inf})}} \tag{4}$$

By deriving this function, the solution of the logistic differential equation (supplementary equation (5)) could be obtained. As a result, $C(t_{inf})$ is subject to the condition $C(t_{inf}) = C_{max}/2$ due to the existing point symmetry of the S-shaped curve:

$$\frac{dC}{dt} = kC\left(1 - \frac{C}{C_{max}}\right) \tag{5}$$

Whereas Odenweller et al. (2022)[54]'s model idea was based on this differential equation (supplementary equation (6)). The resulting adapted model converted C_{max} into a time-dependent demand pull $C_{max(t)}$ and discretised the differential equation, where t denotes the time in years and b the annual growth rate b = ek - 1:

$$C_{t+1} = C_t + bC_t\left(1 - \frac{C_t}{C_{t,max}}\right) \tag{6}$$

We then drew a sample (N = 1000) for the base case and the case with enhanced policy, in each case separately for the corresponding initial capacities in 2030, and for the annual growth rate b of the baseline and sensitivity growth rates using the Monte Carlo simulation. This allowed us to use the values obtained in the adjusted diffusion equation (supplementary equation (4)). Furthermore, the presented model improved numerical accuracy by using a quarterly time resolution with a quarterly growth rate. This growth rate was defined by $b_q = (1 + b)^{1/4} - 1$. Like Odenweller et al. [1], we found no noticeable influence on the results by further increasing the temporal resolution.

Supplementary Table 6. Overview of uncertain parameters for the base case and the case with additional enhanced policy as well as the considered policy levers regarding the baseline emergence growth rates of LNG and wind energy.

<table>
<tr><td rowspan="5"></td><td colspan="15">Uncertain parameters</td></tr>
<tr><td colspan="11"></td><td colspan="4">Policy levers</td></tr>
<tr><td colspan="3">Initial capacity (2030)</td><td colspan="4">Emergence growth rate (LNG)</td><td colspan="4">Emergence growth rate (wind energy)</td><td>i) Short-term capacity (factor)</td><td>ii) Demand anticipation</td><td colspan="2">iii) 2050 DAC market size</td></tr>
<tr><td>Min</td><td>Mean</td><td>σ</td><td>Min</td><td>Mean</td><td>Max</td><td>σ</td><td>Min</td><td>Mean</td><td>Max</td><td>σ</td><td colspan="2"></td><td>Min. Value</td><td>Max. Value</td></tr>
<tr><td colspan="3">[$MtCO_2$ yr^{-1}]</td><td colspan="4">[% yr^{-1}]</td><td colspan="4">[% yr^{-1}]</td><td>[-]</td><td>[years]</td><td colspan="2">[$GtCO_2$ yr^{-1}]</td></tr>
<tr><td>Base case</td><td>4.8</td><td>8.8</td><td>0.95</td><td rowspan="2">0.0</td><td rowspan="2">3.8</td><td rowspan="2">6.8</td><td rowspan="2">1.0</td><td rowspan="2">0.0</td><td rowspan="2">20.0</td><td rowspan="2">42.0</td><td rowspan="2">6.3</td><td>1.0</td><td>5.0</td><td>0.0</td><td>5.1</td></tr>
<tr><td>Case with enhanced policy</td><td>48.0</td><td>88.0</td><td>9.5</td><td>10.0</td><td>15.0</td><td>1.79</td><td>5.1</td></tr>
</table>

Supplementary Notes

The role of cost in DAC diffusion

Cost is among the most critical and uncertain determinants of DAC diffusion. The long-term demand for DAC will depend heavily on its ability to become cost-competitive with other carbon dioxide removal (CDR) options, such as BECCS, enhanced weathering, or afforestation, and with alternative CO_2 sources for utilization or storage. Cost trajectories for DAC are subject to considerable uncertainty. At the gigaton scale, Sievert et al.[27] found a range of DAC costs between $226–$544 for liquid solvent DACCS, $281–$579 for solid sorbent DACCS, and $230–$835 for CaO ambient weathering DACCS. Whether the higher ($835) or lower ($230) end of these ranges materializes will strongly affect the pace of deployment of DAC, since it will directly impact its competitiveness against other CDR and mitigation technologies.

Our model does not explicitly simulate cost dynamics. Instead, cost effects are implicitly captured through the selection of technology analogs, the long-term DAC demand, and the 2030 capacity. First, the analogs were chosen based on complexity and modularity, which are also two key determinants of learning and cost reduction potential[26,27]. Technologies that are modular and less complex, such as wind energy, have historically exhibited faster cost declines and steeper diffusion curves. Higher cost reductions, in turn, are drivers of higher demand, and thus faster adoption. If DAC were to be closer to our more optimistic technology analog (wind energy), this could also reflect into a faster cost reduction and the stimulation of higher demand. Finally, initial 2030 capacity may also be affected by cost reductions. If cost reductions do not materialize in the next few years, investors may lose confidence in future DAC adoption, and they may shy away from novel DAC projects. Our policy scenario, where initial DAC capacity is boosted by a factor 10, could reflect a scenario where, for example, a technological breakthrough would enable lower-than-expected DAC costs, leading to a sudden wave of investments in new DAC projects.

The rapid diffusion of photovoltaics, for example, was enabled not only by inherent modularity but also by massive policy-driven investment and market creation. The same feedback between policy, learning, and adoption will be essential for DAC. Consequently, our results should be interpreted as conditional on the assumption that DAC achieves comparable cost learning to these analogs. Without substantial cost reductions, below approximately 200–300 USD/tCO_2, the scale-up trajectories explored in our scenarios would likely be unattainable.

Supplementary Figures

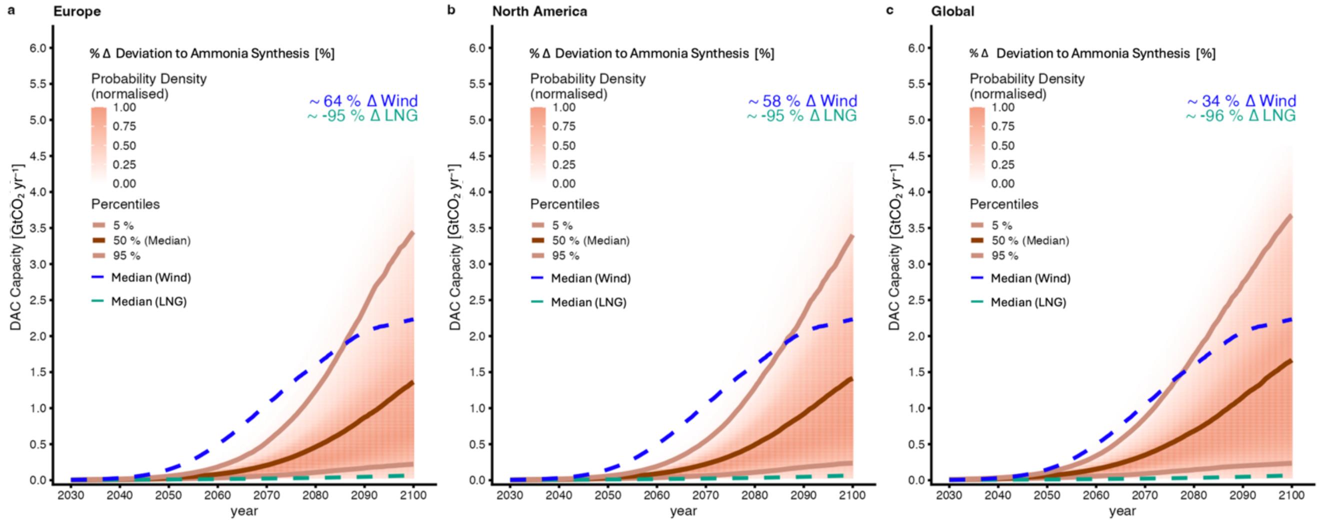

Supplementary Fig. 6. **Regional DAC diffusion feasibility space until 2100.** DAC deployment until 2100 under growth rates of ammonia synthesis, compared with the median deployment paths of the optimistic and pessimistic growth scenarios for wind (blue) and LNG (green) for **(a)** North America, **(b)** Europe and **(c)** the global level.

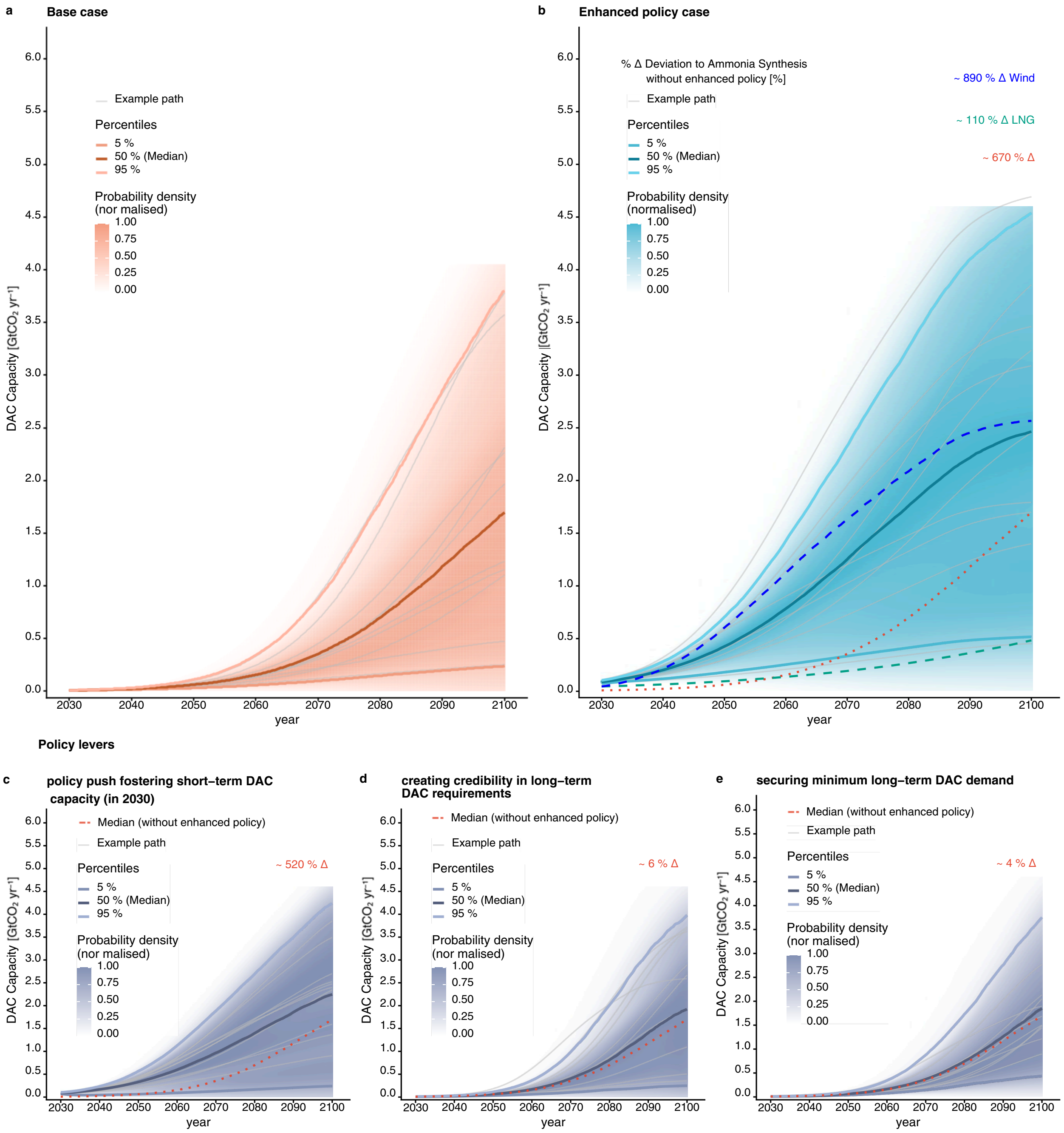

Supplementary Fig. 7. **Probability feasibility space for achieving regional DAC deployment by 2100 globally, based on ammonia synthesis growth rates.** (**a**) Scenarios without policy support (base case) and (**b**) with policy support scenarios. Dashed lines compare optimistic (wind, blue) and pessimistic (LNG, green) analog growth rate sensitivities. Policy scenarios include: (**c**) a policy push to accelerate initial DAC capacity by 2030, (**d**) measures to establish credible long-term DAC demand, and (**e**) policies to secure minimum long-term demand. The color shading indicates the annual probability density (determined from the uncertainty propagation of the initial capacity in 2030 and the emergence growth rate), with grey lines showing example growth paths, representing the broad spectrum of possible outcomes. The deviation to the base case (% Δ) was rounded to the nearest 5th or 10th for all plots in this Figure.

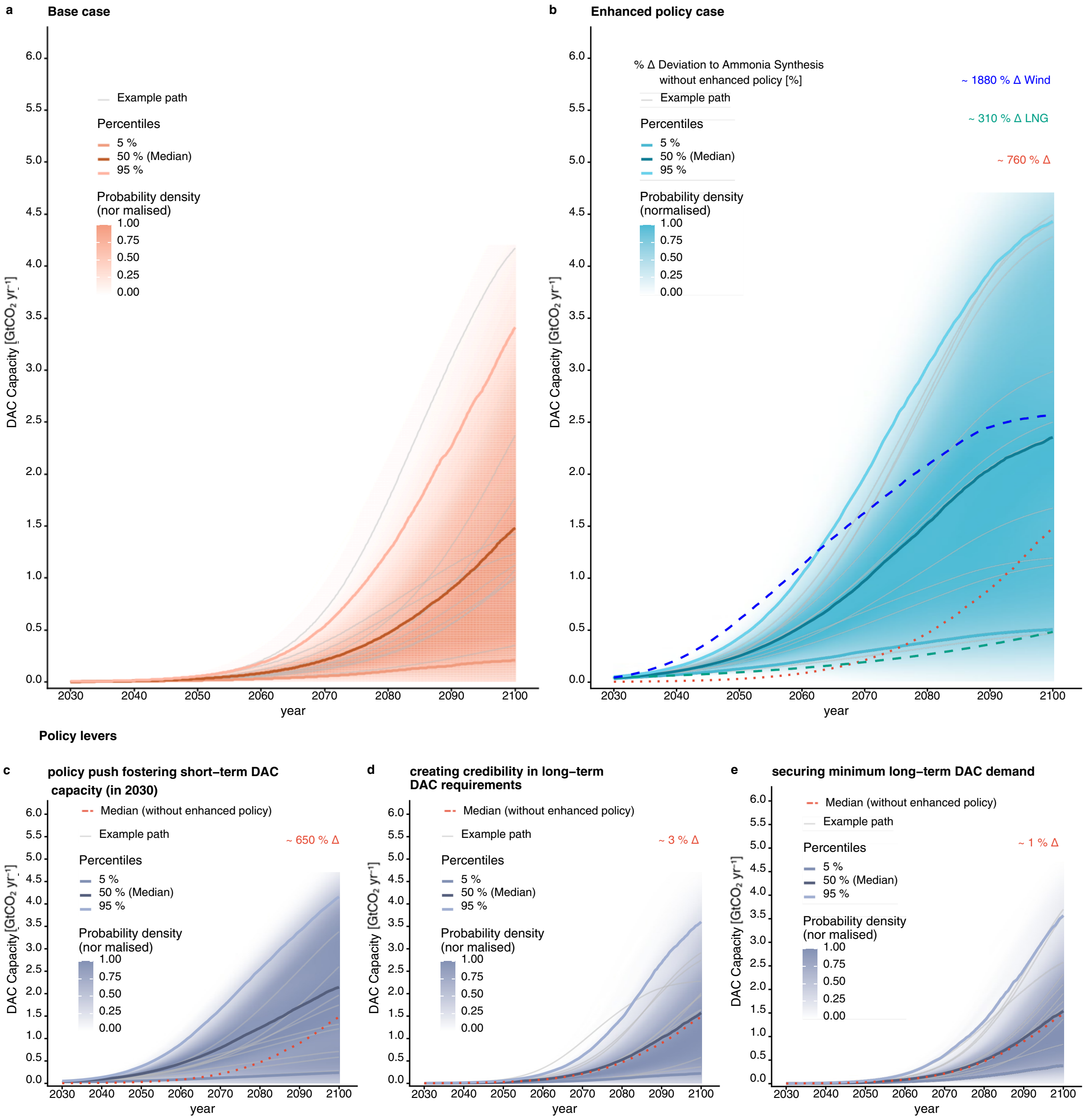

Supplementary Fig. 8. **Probability feasibility space for achieving regional DAC deployment by 2100 in Europe, based on ammonia synthesis growth rates.** (**a**) Scenarios without policy support (base case) and (**b**) with policy support scenarios. Dashed lines compare optimistic (wind, blue) and pessimistic (LNG, green) analog growth rate sensitivities. Policy scenarios include: (**c**) a policy push to accelerate initial DAC capacity by 2030, (**d**) measures to establish credible long-term DAC demand, and (**e**) policies to secure minimum long-term demand. The color shading indicates the annual probability density (determined from the uncertainty propagation of the initial capacity in 2030 and the emergence growth rate), with grey lines showing example growth paths, representing the broad spectrum of possible outcomes. The deviation to the base case (% Δ) was rounded to the nearest 5th or 10th for all plots in this Figure.

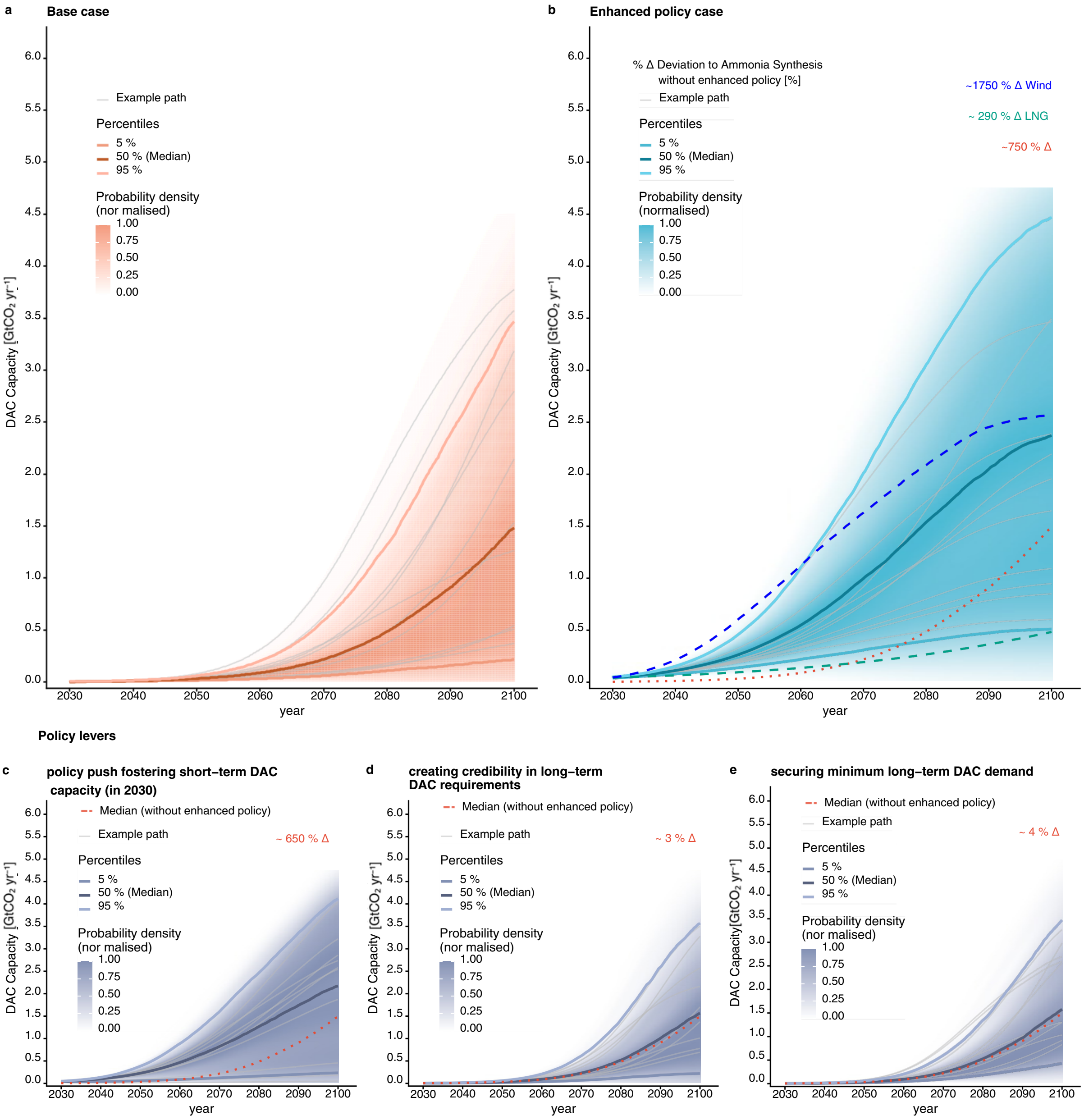

Supplementary Fig. 9. **Probability feasibility space for achieving regional DAC deployment by 2100 in North America, based on ammonia synthesis growth rates**. (**a**) Scenarios without policy support (base case) and (**b**) with policy support scenarios. Dashed lines compare optimistic (wind, blue) and pessimistic (LNG, green) analog growth rate sensitivities. Policy scenarios include: (**c**) a policy push to accelerate initial DAC capacity by 2030, (**d**) measures to establish credible long-term DAC demand, and (**e**) policies to secure minimum long-term demand. The color shading indicates the annual probability density (determined from the uncertainty propagation of the initial capacity in 2030 and the emergence growth rate), with grey lines showing example growth paths, representing the broad spectrum of possible outcomes. The deviation to the base case (% Δ) was rounded to the nearest 5th or 10th for all plots in this Figure.

Supplementary References

1. Odenweller, A., Ueckerdt, F., Nemet, G. F., Jensterle, M. & Luderer, G. Probabilistic feasibility space of scaling up green hydrogen supply. *Nat Energy* **7**, 854–865 (2022).
2. Meade, N. & Islam, T. Modelling and forecasting the diffusion of innovation – A 25-year review. *International Journal of Forecasting* **22**, 519–545 (2006).
3. Rogers, E. M. Diffusion of Innovations. *JPharmSci* **52**, 612 (1963).
4. Schilling, M. A. & Esmundo, M. Technology S-curves in renewable energy alternatives: Analysis and implications for industry and government. *Energy Policy* **37**, 1767–1781 (2009).
5. Li, Y. & Qin, S. Comparative Study on the Developmental Stages of Global CCS Technology Based on the S-Curve Model. *Energy RESEARCH LETTERS* **2**, (2021).
6. Zielonka, N., Wen, X. & Trutnevyte, E. Probabilistic projections of granular energy technology diffusion at subnational level. *PNAS Nexus* **2**, pgad321 (2023).
7. Nemet, G., Zipperer, V. & Kraus, M. The valley of death, the technology pork barrel, and public support for large demonstration projects. *Energy Policy* **119**, 154–167 (2018).
8. Fasihi, M., Efimova, O. & Breyer, C. Techno-economic assessment of CO2 direct air capture plants. *Journal of Cleaner Production* **224**, 957–980 (2019).
9. Arthur, W. B. *Increasing Returns and Path Dependence in the Economy*. (University of Michigan Press, 1994).
10. IEA. Tracking Direct Air Capture. (2023).
11. Ozkan, M., Nayak, S. P., Ruiz, A. D. & Jiang, W. Current status and pillars of direct air capture technologies. *iScience* **25**, 103990 (2022).
12. Smith, S. M. *et al. The State of Carbon Dioxide Removal - 2nd Edition*. (2024).
13. IEA. CCUS Projects Database. https://www.iea.org/data-and-statistics/data-product/ccus-projects-database (2024).
14. Bisotti, F., Hoff, K. A., Mathisen, A. & Hovland, J. Direct Air capture (DAC) deployment: A review of the industrial deployment. *Chemical Engineering Science* **283**, 119416 (2024).

15. IEA. *Direct Air Capture, A Key Technology for Net Zero*. https://iea.blob.core.windows.net/assets/78633715-15c0-44e1-81df-41123c556d57/DirectAirCapture_Akeytechnologyfornetzero.pdf (2022).

16. Yugo, M. & Soler, A. A look into the role of e-fuels in the transport system in Europe (2030–2050). *Concawe Review* **28**, (2019).

17. Nemet, G., Greene, J., Müller-Hansen, F. & Minx, J. C. Dataset on the adoption of historical technologies informs the scale-up of emerging carbon dioxide removal measures. *Commun Earth Environ* **4**, 1–10 (2023).

18. Zurbriggen, T. Feasibility of DAC deployment by 2050: Short-term action is key for gigaton-scale Direct Air Capture by 2050. Zenodo https://doi.org/10.5281/zenodo.18526803 (2026).

19. Roberts, C. & Nemet, G. Lessons for scaling direct air capture from the history of ammonia synthesis. *Energy Research & Social Science* **117**, 103696 (2024).

20. Energy Institute. *Statistical Review of World Energy*. https://www.energyinst.org/statistical-review (2023).

21. IEA. *Net Zero by 2050 - A Roadmap for the Global Energy Sector*. www.iea.org/t&c/ (2021).

22. Fritzeen, W. E. *et al.* Integrated Assessment of the Leading Paths to Mitigate CO2 Emissions from the Organic Chemical and Plastics Industry. *Environmental Science and Technology* **57**, 20571–20582 (2023).

23. Cherp, A., Vinichenko, V., Tosun, J., Gordon, J. A. & Jewell, J. National growth dynamics of wind and solar power compared to the growth required for global climate targets. *Nat Energy* **6**, 742–754 (2021).

24. Hansen, J. P., Narbel, P. A. & Aksnes, D. L. Limits to growth in the renewable energy sector. *Renewable and Sustainable Energy Reviews* **70**, 769–774 (2017).

25. Lowe, R. J. & Drummond, P. Solar, wind and logistic substitution in global energy supply to 2050 – Barriers and implications. *Renewable and Sustainable Energy Reviews* **153**, 111720 (2022).

26. Malhotra, A. & Schmidt, T. S. Accelerating Low-Carbon Innovation. *Joule* **4**, 2259–2267 (2020).

27. Sievert, K., Schmidt, T. S. & Steffen, B. Considering technology characteristics to project future costs of direct air capture. *Joule* **8**, 979–999 (2024).

28. Edwards, M. R. *et al.* Modeling direct air carbon capture and storage in a 1.5 °C climate future using historical analogs. *Proceedings of the National Academy of Sciences* **121**, e2215679121 (2024).

29. Smith, S. M. *et al. The State of Carbon Dioxide Removal - 1st Edition*. http://dx.doi.org/10.17605/OSF.IO/W3B4Z (2023) doi:10.17605/OSF.IO/W3B4Z.

30. IEA. International cooperation in clean energy research and innovation. (2023).

31. Kazemifar, F. A review of technologies for carbon capture, sequestration, and utilization: Cost, capacity, and technology readiness. *Greenhouse Gases: Science and Technology* **12**, 200–230 (2022).

32. Grubb, M., Drummond, P. & Hughes, N. THE SHAPE AND PACE OF CHANGE IN THE ELECTRICITY TRANSITION: Sectoral dynamics and indicators of progress. *UCL Institute for Sustainable Resources* https://www.wemeanbusinesscoalition.org/wp-content/uploads/2020/10/Shape-and-Pace-of-Change-in-the-Electricity-Transition-1.pdf (2020).

33. Beaumont, M. L. L. Making Direct Air Capture Affordable; Technology, Market and Regulatory Approaches. *Front. Clim., Sec. Carbon Dioxide Removal* **4**, (2022).

34. Coffman, D. & Lockley, A. Carbon dioxide removal and the futures market. *Environ. Res. Lett.* **12**, 015003 (2017).

35. McQueen, N. *et al.* A review of direct air capture (DAC): scaling up commercial technologies and innovating for the future. *Prog. Energy* **3**, 032001 (2021).

36. Bachorz, C., Verpoort, P. C., Luderer, G. & Ueckerdt, F. Exploring techno-economic landscapes of abatement options for hard-to-electrify sectors. *Nat Commun* **16**, 3984 (2025).

37. Brazzola, N., Meskaldji, A., Tröndle, T., Patt, A. & Moretti, C. Synthetic fuels may be a cheaper way to achieve climate-neutral aviation. *Nature Communications* ((In review)).

38. Becattini, V., Gabrielli, P. & Mazzotti, M. Role of Carbon Capture, Storage, and Utilization to Enable a Net-Zero-CO2-Emissions Aviation Sector. *Ind. Eng. Chem. Res.* **60**, 6848–6862 (2021).

39. Gabrielli, P., Gazzani, M. & Mazzotti, M. The Role of Carbon Capture and Utilization, Carbon Capture and Storage, and Biomass to Enable a Net-Zero-CO2 Emissions Chemical Industry. *Ind. Eng. Chem. Res.* **59**, 7033–7045 (2020).

40. Union, E. REGULATION (EU) 2023/2405 OF THE EUROPEAN PARLIAMENT AND OF THE COUNCIL of 18 October 2023 on ensuring a level playing field for sustainable air transport (ReFuelEU Aviation), ANNEX 1(f). (2023).

41. Schenuit, F. & Aragones, M. P. Broadening the carbon removal debate beyond the ETS. *Euractiv* https://www.euractiv.com/section/eet/opinion/broadening-the-carbon-removal-debate-beyond-the-ets/ (2024).

42. Johnstone, I., Fuss, S., Walsh, N. & Höglund, R. Carbon markets for carbon dioxide removal. *Climate Policy* **0**, 1–8 (2025).

43. Arning, K. *et al.* Same or different? Insights on public perception and acceptance of carbon capture and storage or utilization in Germany. *Energy Policy* **125**, 235–249 (2019).

44. Lutzke, L. & Árvai, J. Consumer acceptance of products from carbon capture and utilization. *Climatic Change* **166**, 15 (2021).

45. Brazzola, N., Moretti, C., Sievert, K., Patt, A. & Lilliestam, J. Utilizing CO2 as a strategy to scale up Direct Air Capture may face fewer short-term barriers than directly storing CO2. *Environ. Res. Lett.* https://doi.org/10.1088/1748-9326/ad3b1f (2024) doi:10.1088/1748-9326/ad3b1f.

46. Schleussner, C.-F. *et al.* Overconfidence in climate overshoot. *Nature* **634**, 366–373 (2024).

47. IEA. *Net Zero by 2050 – Analysis*. https://www.iea.org/reports/net-zero-by-2050 (2021).

48. IEA. Rapid progress of key clean energy technologies shows the new energy economy is emerging faster than many think. (2023).

49. Burkardt, J. *The Truncated Normal Distribution*. (2023).

50. Abdulla, A., Hanna, R., Schell, K. R., Babacan, O. & Victor, D. G. Explaining successful and failed investments in U.S. carbon capture and storage using empirical and expert assessments. *Environmental Research Letters* **16**, 14036 (2020).
51. Kazlou, T., Cherp, A. & Jewell, J. Feasible deployment trajectories of carbon capture and storage compared to the requirements of climate targets. https://doi.org/10.21203/rs.3.rs-3275673/v1 (2023) doi:10.21203/rs.3.rs-3275673/v1.
52. Lambert, M. EU Hydrogen Strategy – A case for urgent action towards implementation. 4 (2020).
53. Greene, J., Nemet, G., Hammersmith, A. & Zaiser, A. The Historical Adoption of TeCHnology (HATCH) Dataset. https://cdr.apps.ece.iiasa.ac.at/story/hatch/ (2023).
54. Odenweller, A. Climate mitigation under S-shaped energy technology diffusion: Leveraging synergies of optimisation and simulation models. *Technological Forecasting and Social Change* **178**, 121568 (2022).
