# Supplementary material for: Short-term action is key for gigaton-scale Direct Air Capture by 2050
Source: Nat Commun. 2026 May 9;17:6184. doi: 10.1038/s41467-026-72691-3 (PMC13370031; doi:10.1038/s41467-026-72691-3)
Supplement: Supplementary file 2 — Transparent Peer Review file [file 41467_2026_72691_MOESM2_ESM.pdf]

nature portfolio

Peer Review File

Short-term action is key for gigaton-scale Direct Air Capture by 2050

Corresponding Author: Dr Nicoletta Brazzola

This file contains all reviewer reports in order by version, followed by all author rebuttals in order by version.

Version 0:

Reviewer comments:

Reviewer #1

(Remarks to the Author)
In this study, the authors examine the feasibility of achieving "climate-relevant" levels of DAC deployment by mid-century using a probabilistic technology diffusion model. This approach incorporates several key factors, including (1) initial deployment capacity, (2) the credibility of long-term demand, and (3) minimum demand security through an assured long-term market size. Their findings suggest that, in the absence of policy support—particularly near-term demand and initial capacity—DAC deployment is unlikely to exceed the megaton scale. However, with the application of targeted policy levers, higher levels of deployment may become achievable. While the paper addresses an important and timely topic—highly relevant for policymakers and researchers engaged in the CDR and DAC space—the current version does not yet fully communicate its key messages or reflect the depth of effort and innovation behind the work. Below are a few comments that, if addressed, could significantly strengthen the manuscript.

- Determining a climate-relevant level of DAC deployment is inherently uncertain, as it depends on multiple interrelated factors—including the overall demand for CDR (which is shaped by mitigation ambition, residual emissions, and post-net-zero carbon debt) and the relative competitiveness of DAC compared to other CDR methods. Even within the IPCC AR6 scenarios and across various individual IAM studies, there are pathways that achieve Paris-aligned targets with minimal reliance on DAC (see Prütz et al., 2023 for broader uncertainties around the demand for CDR, and Motlaghzadeh et al., 2023 for DAC-specific uncertainty). The authors appear to adopt a target of 1 $GtCO_2$/year by 2050, likely based on mid-range values from 1.5°C or 2°C scenarios in the AR6 database. However, the paper would benefit from a more explicit acknowledgment of the deep uncertainty surrounding future DAC demand. Without this, the paper risks implying that failing to implement strong DAC policy today will necessarily lead to missing climate targets—a conclusion not supported by the broader literature. Although some discussion exists in the Supplementary Information, the main text should clearly articulate the rationale behind selecting 1 $GtCO_2$/year as the benchmark.

- In lines 44 to 47, when discussing the three key policy levers, please include a reference to Table 1 so that readers can easily access the definitions. Additionally, it would be helpful to provide a bit more explanation of the second and third levers.

- Roberts and Nemet (2023) examined analogous technologies and concluded that ammonia synthesis may be a suitable analogue for high-temperature (liquid solvent) DAC, but not for all DAC variants. While your main text does not currently make this distinction, it would be important to clarify that this analogy applies specifically to high-temperature DAC. The technological differences between high- and low-temperature DAC variants are substantial and may limit the generalizability of such analogies. Similarly, CCS, LNG, and ammonia synthesis have primarily been proposed as analogues for high-temperature DAC systems. Additionally, as noted in the Supplementary Information, please consider including in the main text that wind was selected not only to represent the optimistic scenario but also as an analogue for solid sorbent DAC.

- In the main section, I recommend elaborating on the statement in lines 95–96: "DAC would need to follow the growth rates of fast-diffusing technologies." This phrasing is not very intuitive on its own. It would be helpful for the authors to clarify what characterizes such technologies—what key features or enabling conditions allowed them to achieve rapid diffusion. Referring to specific examples or mechanisms, perhaps drawing on insights from the Roberts and Nemet paper, would strengthen the argument and provide useful context for policymakers.

- While I acknowledge the methodological novelty of applying a probabilistic technology diffusion model to DAC, I have some concerns about the significance of the contribution as currently presented. The model—originally applied to green hydrogen scale up by Oddenweller et al. (2022)—is indeed distinct from the approaches used by Nemet et al. (2023) and the IAM-based analysis by Edwards et al. (2023). However, the implications of these methodological differences are not clearly articulated. For example, your results indicate that policy push and initial capacity account for a dominant share of the diffusion impact (~730%), while the remaining factors contribute only about 5%—effects that are presumably not captured in standard IAMs and may be uniquely represented in your model. That said, similar insights also emerge in Nemet et al. (2023), where the authors highlight that "long-term deployment of novel CDR is sensitive to developments in the formative phase," and specifically for DAC, they note that achieving median growth across scenarios would require building approximately ninety 1 $MtCO_2$/year plants by 2040. I suggest the authors elaborate more clearly on the unique features of their methodology—presumably the same as in Oddenweller et al. (2022)—and explicitly discuss how their findings complement or diverge from these related studies. This would help clarify the paper's added value and better position its contribution within the broader literature.

- In lines 53–54 of the SI, the authors state that initial capacity, emergence growth rate, and demand pull are independent. I am not entirely convinced this assumption holds. Are these factors truly independent? For instance, wouldn't initial capacity influence both the growth rate and the strength of future demand pull? If many early DAC projects fail or encounter significant socio-environmental challenges, wouldn't that negatively affect investor confidence, slow growth, or dampen market demand? I recommend that the authors clarify the rationale behind this assumption and discuss any potential interdependencies among these factors.

- Please consider adding the distribution parameters (e.g., mean) of the initial capacity in both the baseline and policy scenarios to the main paper—potentially in Table 1—as they currently appear to be available only in the SI. This is very important to mention in the main text, as you found this policy lever the most influential one.

- Regarding the initial capacity assumptions in the baseline and technology policy scenarios: I appreciate the effort to gather historical and projected DAC deployment data to inform the baseline scenario—this is a valuable contribution. However, I am concerned about the realism of using a capacity factor of 10, which results in a minimum of 48 $MtCO_2$/a, a mean of 88 $MtCO_2$/a, and a maximum of 760 $MtCO_2$/a (I am more concerned about this) by 2030. Could you please clarify the rationale for selecting a capacity factor of 10?

Some additional general comments:
• If space permits, it would be helpful to reference the concept of analogous technologies in the abstract, ideally near the phrase "based on its characteristics alone."
• Some references appear to be duplicated (e.g., references 13 and 29). Please review the reference list for consistency and remove any redundancies.

(Remarks on code availability)

Reviewer #2

(Remarks to the Author)
The manuscript presents the results of a simple logistic diffusion model applied to direct air capture technology. The goal was to examine the feasibility for DAC technologies to scale up to a gigaton level. The model uses three factors: 1. Initial capacity, 2. Growth rate and 3. Demand. The authors concluded that scaling DAC to a Gt level would be challenging and front-loading the expansion would be critical to achieve the goal. While the research question is an important one, I have a number of concerns against its publication as a brief communication article.

First, the model is undoubtedly simple, which perhaps makes it useful for what-if scenario exercises and thought-experiments. However, I doubt whether it can be used as the basis for such an important policy decision. It ignores relevant technological complexities necessary for understanding possible technological and economic trajectories of DAC. There are multiple core technologies in DAC that use a wide variety of physicochemical and thermodynamic principles, and a considerable share of the scaling challenges hinges upon our ability address those specific physics, chemistry, and engineering challenges. The manuscript seems to suggest, quite erroneously in my humble opinion, that one does not need to understand those technical details to reliably predict the future trajectories of a technology. I do acknowledge that learning curves have successfully demonstrated that certain projections can benefit from a very high-level abstraction—one needs not understand how integrated circuits are made to predict its cost trajectory over capacity growth. However, even for those mass-produced commodities, logistic models are known to be highly sensitive to initial conditions and proven to be less useful for nascent technologies.

Second, the authors seem to make a very strong policy recommendation, despite the model's simplicity and inherent limitations and uncertainties. It is my opinion that the model is not worth beyond thought-experimentation and what-if scenario exercises. I believe that the level of confidence in recommending a policy of this gravity should be matched with a lot more rigor and fidelity of the model.

Third, cost of mitigation is central to the question of scale. Unless I missed, I didn't find a meaningful discussion on the role

of cost in modeling the future trajectories of DAC, or why it can be ignored.

Based on these points, I suggest the authors to consider rewriting the manuscript as a what-if scenario exercise, soften the conclusion/policy if recommendations, and discuss the limitations of using the current model.

(Remarks on code availability)

Reviewer #3

(Remarks to the Author)
As the authors have indicated, this paper is mainly premised on the findings of two existing papers:
1- "Probabilistic feasibility space of scaling up green hydrogen supply" by Odenweller et al.
2- "Lessons for Direct Air Capture from the History of Nitrogen Synthesis" by Roberts et al.
By combing the claim of paper #1 with paper #2's methodology, this paper offers a new projection for DAC growth under different policy scenarios.

The paper is well-written, and it is clear and understandable. My main concern is about how policy support and demand commitment are introduced to the model. I think the authors should do a much better job of investigating DAC-specific policies and demand side pull:

1- Some of these issues of course go back to the original paper #2 and the way it compares DAC to nitrogen synthesis. The authors of the paper have been clear however, that "Present conditions for DAC do not resemble those and thus growth at that rate is unlikely." therefore, it is surprising to see that this paper takes the growth rate of ammonia synthesis as the "baseline" growth rate for DAC. The baseline is the value that there is existing evidence for it while this baseline rate is not even supported by the reference paper to be used for DAC.

2- The way the maximum DAC demand is calculated (SI_Table 5) is very questionable. The main justification for having DAC (in contrast to CCU/S) is to have it as DACS, to create negative emissions and to reach net zero. Using DAC as DACU to create synthetic fuel does not make any climate sense or even economic/engineering sense. If the goal is just to get carbon for synthetic fuels and not for negative emission purposes, why should you get it from ambient air when you can get it much cheaper and easier from the point source?

4- This leads me to the next point about carbon market. The term "net-negative" is only mentioned once in the opening paragraph and has been never referred to afterwards. It is very surprising/disappointing to see that a scientific paper on DAC is so silent about the need for negative emissions and the whole discussions around VCM, Paris Agreement article 6 and so on. In my opinion, these are the real market pull levers to investigate and not the synthetic carbon market whose real contribution to net-zero is very debatable.

5- In conclusion, I see two ways for this paper to go: either change the focus and apply the methodology of paper #1 to a more straightforward concept of carbon capture (CCU) where you would be able to justify the use of SI_Table 5 values, or if you want to stick to DAC (and use paper #2 analogue) do it in a correct way and bring in deeper discussions on the viability of carbon markets and other demand-side levers.

(Remarks on code availability)

Version 1:

Reviewer comments:

Reviewer #1

(Remarks to the Author)
The authors have addressed all of my comments, and I believe the manuscript is now ready for publication, provided that it meets the editorial and formatting requirements of Nature Communications. I would like to thank the authors for their detailed and thoughtful responses. My main concerns—particularly regarding the novelty and contribution of the study, as well as the clarification of key assumptions—have been satisfactorily addressed.

(Remarks on code availability)

Reviewer #2

(Remarks to the Author)
I find the authors’ revisions, following the largely unanimous recommendations of the three reviewers, to be substantial. However, I remain concerned that the fundamental hypothesis underlying the manuscript has not been sufficiently corroborated. The usefulness of the modeling work and its results hinges on the statement that:
“[...] in the long run, technology deployment often follows a logistic curve.”

The manuscript's question is even more specific: IF DAC follows the historical trajectories of "ammonia synthesis and liquefied natural gas". Not only are there potentially hundreds or thousands of technologies with differing historical scale-up patterns that could have been used to address such "what-if" scenarios, but I also believe that the study suffers from a conceptual flaw—a category mistake.
Specifically, the authors conceptualize DAC as a single, homogeneous technology, analogous to "ammonia synthesis and liquefied natural gas". Yet the fact that these diverse processes are all labeled "DAC" does not imply the existence of a technology with a unified set of physicochemical or thermodynamic mechanisms of action. This is precisely the type of error Ryle identifies as a "category mistake" in The Concept of Mind (1949).
In practice, it is difficult to justify that fundamentally different DAC approaches—such as amine solvent capture vs. carbonate mineralization, temperature-swing adsorption vs. humidity-swing adsorption, electrochemical vs. thermal/chemical sorbents, or vacuum-swing vs. cryogenic processes—would altogether follow the same or even similar scale-up trajectories. The term DAC is a convenient abstraction, grouping heterogeneous technologies with a common purpose but vastly different mechanisms. The manuscript's assumption that DAC behaves as a single technology with a distinct, predictable scale-up trajectory is therefore unfounded and untested.
I consider the manuscript a valuable thought experiment that highlights important questions regarding DAC scale-up. At the same time, I am not fully convinced that its current approach and assumptions justify publication in a high-profile, peer-reviewed outlet.

(Remarks on code availability)

Reviewer #3

(Remarks to the Author)
Thanks for revising the manuscript and addressing mine and other reviewers' comments. I think the paper is much improved and its limitations are well explained.
I have only one request and one minor comment:

1- It would be essential to state clearly that this analysis is not based on any techno-economic assessment of DAC technologies, nor does it consider any specific mitigation policy, R&D investment choices, or any other interactions among mitigation options.

2-a minor edit in line 199 is needed: "In [the] absence of serious...".

(Remarks on code availability)

Version 3:

Reviewer comments:

Reviewer #2

(Remarks to the Author)
I thank the authors comprehensive response. I found the responses thoughtful and well balanced. I think that the manuscript deserves to be read and examined by the readership of the journal.

(Remarks on code availability)

Reviewer #3

(Remarks to the Author)
Thank you for revising the manuscript and taking my suggestion into account. I believe that the paper provides a valuable contribution to the discussions around carbon dioxide removal technologies given its clearly stated assumptions and limitations.

(Remarks on code availability)

Open Access This Peer Review File is licensed under a Creative Commons Attribution 4.0 International License, which permits use, sharing, adaptation, distribution and reproduction in any medium or format, as long as you give appropriate credit to the original author(s) and the source, provide a link to the Creative Commons license, and indicate if changes were made.
In cases where reviewers are anonymous, credit should be given to 'Anonymous Referee' and the source.
The images or other third party material in this Peer Review File are included in the article's Creative Commons license, unless indicated otherwise in a credit line to the material. If material is not included in the article's Creative Commons license and your intended use is not permitted by statutory regulation or exceeds the permitted use, you will need to obtain permission directly from the copyright holder.
To view a copy of this license, visit https://creativecommons.org/licenses/by/4.0/

RESPONSE TO REVIEWERS

Short-term action is key for gigaton-scale Direct Air Capture by 2050

Tatjana Zurbriggen[1], Nicoletta Brazzola[1,2*], Adrian Odenweller[3], Falko Ueckerdt[3], Joeri Rogelj[4,5]

We would like to begin by sincerely thanking the reviewers for their thoughtful and constructive comments. We fully appreciate the depth and care of the feedback, and we agree with the vast majority of the points raised. As a result, we have undertaken a substantial revision of the manuscript to better communicate the intent, novelty and limitations of our analysis. Specifically, we now frame the work more explicitly as a *what-if* exercise rather than a predictive outlook, using empirically observed logistic dynamics as a basis for if–then projections under uncertainty. Building on this revised framing, we have (1) toned down and more carefully contextualized our policy implications, explicitly highlighting methodological uncertainties; (2) systematically examined and visualized the variability introduced by different choices of technological analogs, showing how results diverge under this critical assumption; and (3) better placed into context the impact of policy levers to clarify the key drivers of uncertainty, and potential intervention points, in accelerating DAC deployment.

Below, we respond point-by-point to reviewers' comment in red. Changes throughout the manuscript and SI are color-coded as follows:

Reviewer 1 – blue
Reviewer 2 – red
Reviewer 3 – green
Multiple reviewers – orange

REVIEWER COMMENTS

Reviewer #1 (Remarks to the Author):
In this study, the authors examine the feasibility of achieving "climate-relevant" levels of DAC deployment by mid-century using a probabilistic technology diffusion model. This approach incorporates several key factors, including (1) initial deployment capacity, (2) the credibility of long-term demand, and (3) minimum demand security through an assured long-term market size. Their findings suggest that, in the absence of policy support—particularly near-term demand and initial capacity—DAC deployment is unlikely to exceed the megaton scale. However, with the application of targeted policy levers, higher levels of deployment may become achievable. While the paper addresses an important and timely topic—highly relevant for policymakers and researchers engaged in the CDR and DAC space—the current version does not yet fully communicate its key messages or reflect the depth of effort and innovation behind the work. Below are a few comments that, if addressed, could significantly strengthen the manuscript.

We thank the reviewer for their constructive and insightful comments. In response, we have substantially revised the manuscript to sharpen both its framing and contributions. Specifically, we now set up the motivation and positioning of the paper more clearly (l. 34–46), clarify the central messages and takeaways of the study (l. 144–148, l. 173–185), and more explicitly articulate the methodological innovations and advances relative to existing literature (l. 50–66, l.

161-171). Together, these revisions ensure that the depth, novelty, and policy relevance of the study are more clearly communicated.

*Please note that changes in the manuscript to address this reviewer's comments are in **blue**. Changes that addressed this reviewer's comments, but also other comments from other reviewers, are in **orange**.*

Comment 1

Determining a climate-relevant level of DAC deployment is inherently uncertain, as it depends on multiple interrelated factors—including the overall demand for CDR (which is shaped by mitigation ambition, residual emissions, and post-net-zero carbon debt) and the relative competitiveness of DAC compared to other CDR methods. Even within the IPCC AR6 scenarios and across various individual IAM studies, there are pathways that achieve Paris-aligned targets with minimal reliance on DAC (see Prütz et al., 2023 for broader uncertainties around the demand for CDR, and Motlaghzadeh et al., 2023 for DAC-specific uncertainty). The authors appear to adopt a target of 1 $GtCO_2$ /year by 2050, likely based on mid-range values from 1.5°C or 2°C scenarios in the AR6 database. However, the paper would benefit from a more explicit acknowledgment of the deep uncertainty surrounding future DAC demand. Without this, the paper risks implying that failing to implement strong DAC policy today will necessarily lead to missing climate targets—a conclusion not supported by the broader literature. Although some discussion exists in the Supplementary Information, the main text should clearly articulate the rationale behind selecting 1 $GtCO_2$ /year as the benchmark.

Response 1

We appreciate the reviewer's comment on the uncertainties surrounding the long-term demand for DAC and agree that determining a "climate-relevant" level of DAC deployment is inherently uncertain, as it depends on multiple interrelated factors. These factors include overall CDR demand (shaped by mitigation ambition, residual emissions, and post-net-zero carbon debt) and the competitiveness of DAC relative to other CDR methods.

We recognize that we should have been clearer about how we determined the long-term DAC demand, which led to some misunderstandings. Our reference to "gigaton scale" deployment may have given the impression that we use a target of 1 $GtCO_2$/year by 2050. However, this is not the case. Our long-term DAC demand (2050) explicitly incorporates the high level of uncertainty surrounding future demand. We use a full range starting with zero (implicitly assuming high biomass availability, low post-net-zero carbon debt) up to a maximum of 5.1 $GtCO_2$/year. The model then randomly samples a DAC target from this range in each run. The higher end of the range, which is however reached in a very small subset of model runs, is derived from an assumption that 50% of the 2050 demand for durable CDR (from the State of CDR Report, Smith et al., 2024) and 50% of the CO_2 feedstock (from the IEA Net Zero Emissions 2050 scenario) would be delivered via DAC (SI_Table 5 and 6). We steered away from simply relying on the AR6 scenarios since in many of the ensemble models DAC is often not treated with sufficient sectoral and technological granularity, especially as CO_2 feedstock.

We instead disentangle the potential use cases and transparently (and simply) derive a potential role of DAC as CDR and in net-zero industries (esp. plastics and residual fuel production). Note that in addition we study an enhanced policy scenario in which we introduce one policy lever termed "Securing minimum long-term DAC demand". This lever increases the lower end of our DAC 2050 range to 1.79 $GtCO_2$/year (which is 35% of the maximum estimated DAC demand). This number is inspired by the 35% share of electricity-based synthetic aviation fuel (SAF) across EU airports by 2050 (*ReFuelEU aviation)*. The policy lever reflects policy makers creating specific policies to create a substantial long-term demand for DAC (beyond aviation in EU).

These choices, and the rationale for not directly using AR6 scenario DAC capacities, are now clearly explained in the main text. We have revised the main text to: (i) explicitly explain the rationale behind our demand-pull assumptions and the bottom-up estimates of long-term demand (l. 55–58, changes to Table 1, l. 217-222), (ii) avoid that references to the "gigaton scale" are read as a specific DAC target and give more context on how this benchmark emerged (l. 34-39), and (iii) emphasize the deep uncertainty in future DAC deployment trajectories (l. 222-226).

Finally, in revising our discussion of risks associated with "slow" DAC growth, we now frame these not as a risk of missing a predetermined DAC benchmark, but as a risk of creating overreliance on large-scale DAC in future scenarios without sufficient action today to enable such growth (l.139-140, l. 142-148, l.190-195). This reframing, which also partially responds to concerns of reviewer 3, makes clear that the central concern is not that of missing a particular DAC capacity target, but rather that of creating expectations for DAC's contribution to net zero emissions and beyond that may prove infeasible.

Comment 2
- In lines 44 to 47, when discussing the three key policy levers, please include a reference to Table 1 so that readers can easily access the definitions. Additionally, it would be helpful to provide a bit more explanation of the second and third levers.

Response 2
Thank you, we have substantially expanded the description of the policy levers in the Methods section and explicitly referred to Table 1 in the main text (l. 64-66, l. 233-249).

Comment 3
- Roberts and Nemet (2023) examined analogous technologies and concluded that ammonia synthesis may be a suitable analogue for high-temperature (liquid solvent) DAC, but not for all DAC variants. While your main text does not currently make this distinction, it would be important to clarify that this analogy applies specifically to high-temperature DAC. The technological differences between high- and low-temperature DAC variants are substantial and may limit the generalizability of such analogies. Similarly, CCS, LNG, and ammonia synthesis have primarily been proposed as analogues for high-temperature DAC systems. Additionally, as noted in the Supplementary Information, please consider including in the main text that wind

was selected not only to represent the optimistic scenario but also as an analogue for solid sorbent DAC.

Response 3
We thank the reviewer for pointing out the important distinctions between different DAC variants. We agree that the technological differences between high-temperature (liquid solvent) DAC and low-temperature (solid sorbent) DAC are substantial, and that this limits the generalizability of analogies across all DAC types.

In the revised manuscript, we now therefore: (i) explicitly acknowledge the differences between DAC technologies in the main text, clarifying that our use of ammonia synthesis as a baseline growth analogue applies most directly to high-temperature DAC (l. 73-76); (ii) note that wind energy was included not only as an optimistic case, but also as an analogue for solid sorbent DAC (l. 76-78).

At the same time, we have chosen not to assign different growth rates to different DAC types. This decision is motivated by two considerations: first, applying different growth rates would contrast with what often observed in technological transition, namely that a dominant technology design often ultimately prevails; and second, available project data do not consistently resolve DAC type, such that distinguishing robustly between growth pathways for high- and low-temperature variants is not feasible at this stage.

Instead, we now discuss more transparently the uncertainty associated with different technology analogs (also addressing concerns of reviewer 3) (l. 69-71). To further highlight the uncertainties in choosing any single analogue, we have broken down Figure 1 into two complementary perspectives: (i) a figure showing illustrative outcomes under different technological analogs (l.93-111), and (ii) a “policy levers” figure, which emphasizes how policy choices affect outcomes (l. 113-137).

Comment 4
- In the main section, I recommend elaborating on the statement in lines 95–96: “DAC would need to follow the growth rates of fast-diffusing technologies.” This phrasing is not very intuitive on its own. It would be helpful for the authors to clarify what characterizes such technologies—what key features or enabling conditions allowed them to achieve rapid diffusion. Referring to specific examples or mechanisms, perhaps drawing on insights from the Roberts and Nemet paper, would strengthen the argument and provide useful context for policymakers.

Response 4
We thank the reviewer for highlighting that we need to clarify the technological characteristics that rapid diffusion.

Generally, more modular technologies can scale-up more quickly as they enable more frequent iterative learning-by-doing cycles, which facilitates mass production that unlocks cost savings, which again spurs growth due to improved economic competitiveness (Wilson et al., 2020,

https://www.science.org/doi/10.1126/science.aaz8060). In contrast, large one-of-a-kind projects often lead to cost overruns and delays (Flyvbjerg, 2022, How Big Things Get Done).

In the manuscript, we have now elaborated on these technology characteristics (l. 158-160), while respecting the tight word limit.

Comment 5

- While I acknowledge the methodological novelty of applying a probabilistic technology diffusion model to DAC, I have some concerns about the significance of the contribution as currently presented. The model—originally applied to green hydrogen scale up by Odenweller et al. (2022)—is indeed distinct from the approaches used by Nemet et al. (2023) and the IAM-based analysis by Edwards et al. (2023). However, the implications of these methodological differences are not clearly articulated. For example, your results indicate that policy push and initial capacity account for a dominant share of the diffusion impact (~730%), while the remaining factors contribute only about 5%—effects that are presumably not captured in standard IAMs and may be uniquely represented in your model. That said, similar insights also emerge in Nemet et al. (2023), where the authors highlight that "long-term deployment of novel CDR is sensitive to developments in the formative phase," and specifically for DAC, they note that achieving median growth across scenarios would require building approximately ninety 1 $MtCO_2$ /year plants by 2040. I suggest the authors elaborate more clearly on the unique features of their methodology—presumably the same as in Oddenweller et al. (2022)—and explicitly discuss how their findings complement or diverge from these related studies. This would help clarify the paper's added value and better position its contribution within the broader literature.

Response 5

We thank the reviewer for acknowledging our methodological novelty and for encouraging us to explain the unique features of our approach more thoroughly. We would like to highlight the following key advances that our method offers in comparison to Nemet et al. (2023). First, Nemet et al. (2023) do not use a full project database, but only loosely refer to "company announcements" and simply assume a size of 1 MtCO2/yr per announced plant. Second, they don't provide a quantitative estimate of the impact of different policy levers, but only a qualitative comparison using technology analogs. Third, their approach therefore cannot capture different sources of uncertainty in scaling-up DAC. Lastly, and related to the previous point, they don't account for the uncertainty of long-term demand for DAC.

In the revised manuscript, we now highlight these novelties more explicitly (l. 161-171).

Comment 6

- In lines 53–54 of the SI, the authors state that initial capacity, emergence growth rate, and demand pull are independent. I am not entirely convinced this assumption holds. Are these factors truly independent? For instance, wouldn't initial capacity influence both the growth rate and the strength of future demand pull? If many early DAC projects fail or encounter significant socio-environmental challenges, wouldn't that negatively affect investor confidence, slow

growth, or dampen market demand? I recommend that the authors clarify the rationale behind this assumption and discuss any potential interdependencies among these factors.

Response 6
We agree that in the real-world initial capacity, emergence growth rate, and demand pull are not strictly independent. Early project performance and socio-environmental outcomes can clearly influence investor confidence, slow or accelerate growth, and shape perceptions of long-term demand. For example, as has already been observed in the case of green hydrogen, low success rates of project announcements can undermine credibility and slow investment momentum.

In our modelling framework, however, we treat these parameters as independent by design. This is a modelling choice that approximates but simplifies the real-world dynamics. We made this choice for three reasons:

- Analytical clarity: Isolating these factors allows us to identify which of them has the largest impact on DAC diffusion trajectories.
- Consistency with S-curve dynamics: Historically, many technologies have followed logistic (S-curve) deployment pathways despite underlying interdependencies, which suggests that treating the parameters independently can still approximate aggregate growth patterns reasonably well.
- Model implementation: In our model, the emergence growth rate only begins in the initial year at the uncertain initial capacity. This enables us to incorporate near-term DAC project announcements into our analysis, while accounting for the inherent uncertainty. This approach also guarantees that enough DAC capacity is installed to justify applying growth rates for the following scale-up phase. The initial capacity therefore establishes the uncertain starting point, while the subsequent trajectory of the curve is subject to the growth rate, preserving independence in the simulation.

We also acknowledge that project failures, cost overruns, or social challenges can feed back into both growth expectations and long-term demand pull. Importantly, uncertain costs are a critical mediating factor: projects that fail due to excessively high costs not only reduce near-term capacity growth but may also undermine the credibility of DAC as a long-term solution, thereby dampening demand.

We have revised the Methods in the main manuscript text (l. 250-255) and the Supplementary Information (l. 55-65) and added these clarifications to ensure that the assumptions and their limitations are transparent.

Comment 7
Please consider adding the distribution parameters (e.g., mean) of the initial capacity in both the baseline and policy scenarios to the main paper—potentially in Table 1—as they currently appear to be available only in the SI. This is very important to mention in the main text, as you found this policy lever the most influential one.

Response 7
Thanks for this, we expanded Table 1 to include the uncertainty parameters of the three uncertainty parameters in both the base case and the policy case. We have also provided these values in the Supplementary Information (l. 126-129).

Comment 8

- Regarding the initial capacity assumptions in the baseline and technology policy scenarios: I appreciate the effort to gather historical and projected DAC deployment data to inform the baseline scenario—this is a valuable contribution. However, I am concerned about the realism of using a capacity factor of 10, which results in a minimum of 48 $MtCO_2$ /a, a mean of 88 $MtCO_2$ /a, and a maximum of 760 $MtCO_2$ /a (I am more concerned about this) by 2030. Could you please clarify the rationale for selecting a capacity factor of 10?

Response 8

We thank the reviewer for pointing out at these numbers, which indeed were mistakenly included and led to confusion. We model the initial capacity as a truncated normal distribution. For the base scenario, we have a mean of 8.8 $MtCO_2$ /a by 2030, which we truncated at the lower end at 4.4 $MtCO_2$ /a, corresponding to the projects that are already either operational or under construction (using the same approach as in Figure 3a,b of Odenweller et al., 2022). Including all announced plants in our sample results into over 150 $MtCO_2$ /a. Yet, because we model this as a normal distribution, the probability of reaching such high initial capacity is close to zero and, in effect, looking at our Figure 1, you can see that the initial capacity range is below a dozen $MtCO_2$ /a.

In our policy scenario, the increase in capacity by a factor of 10 is intended to reflect an upper-bound, highly ambitious deployment pathway consistent with the rapid expansion of capacity that can occur once commercial deployment takes off in emerging technology sectors. The mean initial capacity 88 $MtCO_2$ /a is still aligned with recent modelling projections of DAC capacity by 2030. For example, the most recent WEO 2024 report has 68 $MtCO_2$ /a removed by DAC in 2030. Moreover, the 2030 global projection the IEA Net Zero Emissions by 2050 Scenario projects DAC technologies capturing more than 85 $MtCO_2$ /a in 2030.

We acknowledge that scaling DAC from a few $MtCO_2$ /a to almost hundred $MtCO_2$ /a by 2030 would require completing by 2030 all of the projects that have only been planned or announced. Because in reality projects often do not materialize, or experience significant delay, this is a clearly optimistic scenario. Yet, for the purpose of our what-if analysis, we believe we could assume a scenario where we see exceptional policy support, unprecedented infrastructure development, and market expansion beyond the current frontrunners.

To improve the clarity of the description of the initial capacity and amend previous mentions of 76 $MtCO_2$ /a, we have revised Table 1 (see response to comment 7) and of the lever description (see response to comment 2 and l. 55-58, 233-239). Moreover, we have added SI_Figure 3.2 to show the distribution of 2030 DAC capacities (l. 145-151 of SI) and amended the description of the initial capacity calculations (l. 126-129 of SI).

Some additional general comments:

• If space permits, it would be helpful to reference the concept of analogous technologies in the abstract, ideally near the phrase "based on its characteristics alone."
• Some references appear to be duplicated (e.g., references 13 and 29). Please review the reference list for consistency and remove any redundancies.

We have added the notion of tehcnological analogs in the abstract (l. 15-16) and reviewed the reference list. Please not that references 13 and 29 in the original manuscript were distinct references but from the same author and the same year.

Reviewer #2 (Remarks to the Author):

The manuscript presents the results of a simple logistic diffusion model applied to direct air capture technology. The goal was to examine the feasibility for DAC technologies to scale up to a gigaton level. The model uses three factors: 1. Initial capacity, 2. Growth rate and 3. Demand. The authors concluded that scaling DAC to a Gt level would be challenging and front-loading the expansion would be critical to achieve the goal. While the research question is an important one, I have a number of concerns against its publication as a brief communication article.

We thank the reviewer for recognizing the importance of the research question. As elaborated in our detailed responses below, our aim with this work is not to predict a specific DAC deployment trajectory, but rather to conduct a structured *what-if* analysis of the conditions under which DAC could plausibly reach climate-relevant scales by mid-century. In response to the reviewer's concerns, we have now made this framing explicit throughout the manuscript, clarified the scope and limitations of our methodological approach, and more carefully contextualized the resulting policy implications to avoid overinterpretation. We believe these revisions improve the clarity and transparency of the manuscript, while maintaining the contribution of offering a structured, empirically informed way of exploring plausible ranges and orders of magnitude for DAC upscaling.

Please note that changes in the manuscript to address this reviewer's comments are in ***red****. Changes that addressed this reviewer's comments, but also other comments from other reviewers, are in* ***orange****.*

Comment 1
First, the model is undoubtedly simple, which perhaps makes it useful for what-if scenario exercises and thought-experiments. However, I doubt whether it can be used as the basis for such an important policy decision. It ignores relevant technological complexities necessary for understanding possible technological and economic trajectories of DAC. There are multiple core technologies in DAC that use a wide variety of physicochemical and thermodynamic principles, and a considerable share of the scaling challenges hinges upon our ability address those specific physics, chemistry, and engineering challenges. The manuscript seems to suggest, quite erroneously in my humble opinion, that one does not need to understand those technical details to reliably predict the future trajectories of a technology. I do acknowledge that learning

curves have successfully demonstrated that certain projections can benefit from a very high-level abstraction—one needs not understand how integrated circuits are made to predict its cost trajectory over capacity growth. However, even for those mass-produced commodities, logistic models are known to be highly sensitive to initial conditions and proven to be less useful for nascent technologies.

Response 1

Thank you for this insightful review. We fully agree, and did not intend to suggest otherwise, that overcoming technical challenges is fundamental for any scale-up, and that such challenges derive directly from the technological, market, and policy characteristics specific to DAC. At the same time, for novel technologies the future evolution of these factors is highly uncertain. This is why, to explore the feasibility space, it is common to adopt the simplifying assumption that emerging technologies will approximate the deployment dynamics of earlier technologies, based on the empirical evidence that, in the long run, technology deployment often follows a logistic curve.

Our study uses these stylized insights into logistic technology growth to build a deliberately simple model for examining the uncertainty of DAC upscaling. While simple, this approach represents the state of the art in diffusion modelling: it is grounded in empirically observed trajectories, improves upon previous DAC diffusion studies that relied on less comprehensive assessments of initial DAC capacity, and crucially incorporates a probabilistic framework. This allows us to vary the initial DAC demand. This step is particularly important given the sensitivity of logistic models to initial conditions. We also circumvented some typical methodological pitfalls such as overfitting (e.g., Baumgärtner et al., 2024, https://doi.org/10.1016/j.joule.2024.07.016; Hansen et al., 2017, https://doi.org/10.1016/j.rser.2016.11.257) by not doing any statistical fitting, since—as the reviewer rightly points out—we are way too early in the diffusion.

We agree, however, that the transparency of our assumptions and the limitations of our approach should be more clearly highlighted to ensure that readers interpret the policy implications with appropriate caution. In response, we have:

- Reframed the study as an *if–then* experiment rather than as a predictive exercise, and avoided any wording that could be misread as a forecast (l. 46-48, 60, 109-111, 146-154).
- Explicitly acknowledged the simplicity and limitations of our approach which however also enables us to avoid the pitfalls of overfitting logistic diffusion models to nascent technologies (l. 50-58, 178-181).
- Clarified the conditional nature of our policy implications, noting that they rely on the assumption that analogies to prior technologies hold and that DAC diffusion can be approximated by logistic growth (l. 178-185).
- Shown the effects of different growth rates from the selection of different technological analogs in a separate figure, such to highlight the uncertainties associated with the selection of an analog (new Figure 1 and l. 94-99, see also Response 3 to Reviewer 1).
- Emphasized the sensitivity to initial conditions and explained how our probabilistic analysis helps to address this (l. 164-165).

Comment 2

Second, the authors seem to make a very strong policy recommendation, despite the model's simplicity and inherent limitations and uncertainties. It is my opinion that the model is not worth beyond thought-experimentation and what-if scenario exercises. I believe that the level of confidence in recommending a policy of this gravity should be matched with a lot more rigor and fidelity of the model.

Response 2

We fully agree that our model is best understood as a scenario-based thought experiment rather than a predictive policy tool. The study was designed to explore *if–then* relationships and, specifically, what rates of diffusion and capacity growth could be achieved *if* DAC were to follow analogous historical technology trajectories under various policy levers.
Also answering the previous comment, we have revised the manuscript to make this framing clearer throughout (l. 46-48, 102-111, 146-154). We now explicitly note that our policy-relevant insights rest on key assumptions namely that (i) the diffusion of DAC follows a logistic growth pattern similar to historical clean technology analogs, and (ii) that technological learning, cost reductions, and policy responses scale comparably (l. 178-181). We also emphasize that, given the nascency of DAC and the simplifications of our model, our findings should be interpreted as illustrative rather than prescriptive (l.181-185).

Comment 3

Third, cost of mitigation is central to the question of scale. Unless I missed, I didn't find a meaningful discussion on the role of cost in modeling the future trajectories of DAC, or why it can be ignored.

Response 3

We fully agree that cost is a central determinant of DAC's long-term scalability and that our simplified model does not explicitly represent cost dynamics.

The demand levers of our model are based on the implicit assumption that demand (i.e., willingness to pay and subsequent investment decisions) would only materialize if DAC can reach cost parity with competing technology – both fossil-based technologies and climate solutions such as CDR methods and other sustainable CO_2 feedstocks. This cost parity can however only be the result of policy interventions, such as carbon taxes, subsidies or minimum quotas. The dependence on in particular DAC-specific policies becomes apparent when comparing today's costs of DAC (500–1000 $/tCO_2$) with current carbon prices (EU ETS <100 $/tCO_2$). In addition, recent long-term estimates of DAC are higher than expected before (Sievert et al., 2024).

When introducing three policy levers in our policy scenario, we do not explicitly track the development of these cost gaps. However, the policy levers vary with respect to how and when cost gaps are closed such that market actors start investing in DAC applications. For example,

the lever “policy push fostering short-term DAC capacity (in 2030)” reflects a subsidy (e.g., via capacity auctioning) that translates into short-term projects – without quantifying the associated policy costs (e.g., subsidy volumes), which is out of scope of this work.

In addition, to acknowledge the important role of cost for DAC diffusion, we have now added a discussion to clarify that, in reality, cost strongly influences diffusion rates through both learning effects and policy feedbacks (e.g., Lilliestam et al., 2017, https://www.nature.com/articles/nenergy201794). We also emphasized that long-term DAC deployment remains highly uncertain and depends critically on its ability to achieve cost competitiveness e.g., with other CDR options and with point-source CO_2 capture. This clarification has been added in the main text (l. 181-184) and discussed in more detail in the new SI section on cost-related uncertainties (l. 425-453).

We also explicitly note, in the SI, that our approach indirectly accounts for cost developments through the choice of technology analogs: since the analogs were selected based on complexity and modularity—parameters empirically linked to learning rates (Malhotra & Schmidt, 2020; Sievert et al., 2024)—the resulting diffusion patterns implicitly reflect technologies that experienced cost declines comparable to what might be expected for DAC.

Based on these points, I suggest the authors to consider rewriting the manuscript as a what-if scenario exercise, soften the conclusion/policy if recommendations, and discuss the limitations of using the current model.

Thank you for your thoughtful suggestions, which we tried to accommodate.

Reviewer #3 (Remarks to the Author):

As the authors have indicated, this paper is mainly premised on the findings of two existing papers:
1- "Probabilistic feasibility space of scaling up green hydrogen supply" by Odenweller et al.
2- "Lessons for Direct Air Capture from the History of Nitrogen Synthesis" by Roberts et al.
By combing the claim of paper #1 with paper #2's methodology, this paper offers a new projection for DAC growth under different policy scenarios.

The paper is well-written, and it is clear and understandable. My main concern is about how policy support and demand commitment are introduced to the model. I think the authors should do a much better job of investigating DAC-specific policies and demand side pull:

Thanks so much for the thoughtful and constructive comments. We particularly appreciate the insights regarding our choice of the technological analog as the baseline scenario and the suggestion to emphasize the critical role of DAC for net-negative emissions. We believe that the concern about DAC demand stems from a misunderstanding of our methodological approach, which was also noted by Reviewer 1 (see Response 1). To address this, we have revised the

manuscript to provide a clearer explanation of our approach and to more explicitly discuss its limitations.

Please note that changes in the manuscript to address this reviewer's comments are in ***green****. Changes that addressed this reviewer's comments, but also other comments from other reviewers, are in* ***orange****.*

Comment 1
Some of these issues of course go back to the original paper #2 and the way it compares DAC to nitrogen synthesis. The authors of the paper have been clear however, that "Present conditions for DAC do not resemble those and thus growth at that rate is unlikely." therefore, it is surprising to see that this paper takes the growth rate of ammonia synthesis as the "baseline" growth rate for DAC. The baseline is the value that there is existing evidence for it while this baseline rate is not even supported by the reference paper to be used for DAC.

Response 1
Thanks, we agree that ammonia synthesis should not be interpreted as a "baseline" growth rate for DAC, given that present conditions for DAC deployment differ substantially from those observed during the historical development of ammonia synthesis. Accordingly, we have removed the term "baseline" throughout the manuscript to avoid implying that there is empirical evidence supporting this specific growth rate (l. 55-58, l. 69-71, l. 73-74, Table 1, l. 102-104, l. 126). The remaining mentions to the "base case" refer to a case without additional policy levers.

In the revised version, we also clarify the uncertainties and "if–then" nature of the analog-based modeling approach, emphasizing that our analysis is exploratory and not predictive (see also Response 1 and 2 to Reviewer 2). The manuscript now explicitly states that the choice of analogs introduces structural uncertainty (l. 67-69, 101-102, Table 1), and that the results should be interpreted as indicative of possible diffusion trajectories under different historical precedents rather than as expectations of actual DAC growth (l. 146-148, 173-175, 184-185).

As described in our Response 3 to Reviewer 1, we have also refined the framing of technology analogues: ammonia synthesis is discussed as being most relevant for high-temperature (liquid solvent) DAC, while wind energy provides a closer parallel for low-temperature (solid sorbent) DAC (l. 69-78). Although we chose not to assign distinct growth rates to different DAC variants—given current data limitations and to preserve the consistency of the scenario-based framework—we now make this rationale explicit in the text. We moreover explicitly state that the choice of ammonia synthesis as the basis for performing the policy-lever analysis is not because the analogy to this technology is a perfect analog for DAC, but rather because it leads to 'middle of the road' growth rates and thus is better placed to perform further what-if analyses than more extreme scenarios.

Finally, to explicitly highlight the uncertainty associated with different technological analogs, we have restructured Figure 1 to present growth trajectories under multiple analogs separately (l. 92-111). We have separated this figure from a complementary "policy levers" figure (Figure 2) that highlights how different policy conditions influence deployment outcomes.

Comment 2
The way the maximum DAC demand is calculated (SI_Table 5) is very questionable. The main justification for having DAC (in contrast to CCU/S) is to have it as DACS, to create negative emissions and to reach net zero. Using DAC as DACU to create synthetic fuel does not make any climate sense or even economic/engineering sense. If the goal is just to get carbon for synthetic fuels and not for negative emission purposes, why should you get it from ambient air when you can get it much cheaper and easier from the point source?

Response 2
We thank the reviewer for raising this important point. In our view it relates to two ongoing debates and we want to respond to both of them:

1) Using point source CO2 instead of DAC.
2) DAC for carbon utilization (DACCU) versus carbon storage (DACCS).

On the first one, we agree that the primary climate rationale for DAC lies in its potential to deliver negative emissions via DACCS and acknowledge that DACCU is an expensive and currently uncertain pathway. Nevertheless, a share of future DAC deployment may also be driven by synthetic hydrocarbon production in sectors where direct electrification or biomass-based alternatives remain constrained or infeasible. Utilizing point-captured (fossil) CO_2 as a feedstock, which the reviewer points to as a more cost-effective option, is not aligned with the concept of net zero emissions, as it is only a delay in fossil emissions, if the product eventually is combusted and releases the captured CO_2 back to the atmosphere. Hence, while fossil point sources could play a bridging role as long as DAC-based or biogenic carbon is scarce, DAC likely needs to be developed early enough to contribute substantially to providing synthetic fuels and feedstocks long term. Biogenic CO_2 feedstock from biomass, on the other hand, is likely to play a large long-term role in aligning alternative fuels, chemicals, and materials with the net zero target. However, many studies have pointed to their limited availability due to competing demand for biomass (e.g., Uludere Aragon et al., 2022 - https://doi.org/10.1029/2023EF003866, Royal Society, 2023 - https://royalsociety.org/-/media/policy/projects/net-zero-aviation/net-zero-aviation-fuels-policy-briefing.pdf).

Regarding the second debate, some stakeholders and analysts argue that DAC should be deployed exclusively as part of DACCS to compensate for hard-to-abate emissions, such as those from aviation fuels. According to this view, offsetting fossil kerosene (or any remaining emissions) through DACCS would render DACCU in aviation – and in any other sector – unnecessary. While DACCS will likely remain cheaper than DACCU (e.g., Bachorz et al., 2025), several arguments suggest that DACCU is still expected to play a substantial role. We briefly highlight three points:

- **Cost considerations.** In aviation, reducing non-CO_2 climate impacts through the use of e-kerosene can be more cost-effective than relying solely on DACCS, when the non-CO_2 warming effects of aviation are taken into account (Brazzola et al., 2025).
- **Robustness and credibility.** A key concern with DACCS – and CDR more broadly – is the risk of delaying emission-reduction efforts. Significant CDR scale-up is already

required to compensate for non-CO_2 emissions alone. DACCU can reduce dependence on large-scale CCS-based CDR, whose rapid deployment remains uncertain, especially given the "lumpy," site-specific nature of CCS storage and transport infrastructure. Supporting both DACCS and DACCU therefore enhances system robustness and strengthens the credibility of climate-mitigation pathways.

- **Current policy trends.** From a descriptive perspective, current policy developments indicate continued support for synthetic fuels, for example under the ReFuelEU regulation, suggesting that DAC-derived fuels are likely to play a role in practice.

Based on these considerations, we regard our assumption that up to 50% of 2050 CO_2 feedstock demand could come from DAC as an extreme, yet useful upper-bound of the future potential demand for DAC. This upper bound is somewhat symmetrical in its 'extremeness' to our assumption of a lower bound by 0 MtCO2 by 2050, corresponding to a scenario where no DAC at all is demanded in 2050. Since the future potential demand for DAC is extremely uncertain, we sample the 2050 demand for our model runs from a homogeneous distribution ranging between 0 and 5.1 $GtCO_2$.

We acknowledge that the manuscript lacked a clear description of the rationale behind our projected 2050 demand range, and a lack of contextualization on the potential future role of DAC. To clarify this aspect, we have:

1. Highlighted the uncertain future role of DAC and DACCU specifically (l. 26-30, Table 1)
2. Expanded the description in the main text of how DAC demand was constructed "bottom up" in the main text (l. 55-58, l. 211-226, l. 240-243, Table 1). See also our Response 3 to Reviewer 1.
3. Expanded the rationale for calculating DAC demand bottom up, distinguishing between DACCS and DACCU (SI l. 200-245). We now provide a fuller discussion of the arguments *for and against* including DACCU in the demand estimate. This includes references to techno-economic assessments supporting the reviewer's view (e.g., Bachorz and Ueckerdt et al.) and counterarguments that highlight existing political and policy momentum (e.g., Brazzola et al., 2024, and the discussions summarized in Bachorz/Ueckerdt et al.).

Comment 3
This leads me to the next point about carbon market. The term "net-negative" is only mentioned once in the opening paragraph and has been never referred to afterwards. It is very surprising/disappointing to see that a scientific paper on DAC is so silent about the need for negative emissions and the whole discussions around VCM, Paris Agreement article 6 and so on. In my opinion, these are the real market pull levers to investigate and not the synthetic carbon market whose real contribution to net-zero is very debatable.

Response 3
We thank the reviewer for this insightful comment and fully agree that achieving net-negative emissions will be essential to returning from potential temperature overshoots of the 1.5C target later in the century. We also share the view that the unique value of DAC lies in its potential for

large-scale atmospheric CO_2 removal, given its comparatively limited biophysical constraints relative to other carbon dioxide removal (CDR) options.

The brevity of the original manuscript (1,500 words) and the 2050 time horizon of our analysis required us to focus primarily on DAC's role in enabling net-zero emissions consistent with 1.5-2C pathways. Nonetheless, we acknowledge that DAC's significance extends well beyond 2050, particularly in facilitating the transition to net-negative emissions in the latter half of the century. We believe the reviewer's interpretation that our discussion of potential CO_2 utilization implied a focus on DACCU rather than DACCS reflects an unintended ambiguity in our framing.

As clarified in Response 2, the inclusion of DACCU-related demand was intended solely as an uncertainty range for possible DAC deployment by 2050-not as an assumption about the long-term structure of the DAC market. We fully agree that, in the long run, DACCS is expected to dominate overall DAC demand, and that policy mechanisms such as Article 6 of the Paris Agreement, the EU ETS, and other compliance carbon markets are likely to be more influential levers than near-term synthetic fuel markets. However, our model 'mimics' these policies and market conditions only via the demand-pull parameters, which is why we have modelled better policy conditions as a enhancement of the minimum long-term DAC demand.

To ensure our intent is clear, we have revised the manuscript accordingly:

1. We now explicitly highlight DAC's potential role in enabling large-scale net-negative emissions, particularly given its fewer biophysical constraints compared to land-based CDR options (l. 25-26, 141-144). We reference Schleussner et al., 2025 (https://www.nature.com/articles/s41586-024-08020-9) to emphasize that sustainably scalable CDR capacities may be largely exhausted by mid-century-underscoring DAC's importance for addressing potential overshoots if its energy intensity declines.
2. We have expanded the discussion of policy and market levers to acknowledge the voluntary carbon market (VCM) as a short-term mechanism to stimulate early deployment, while clarifying that long-term DAC growth will more likely depend on integration into compliance markets (e.g., Article 6, EU ETS, ReFuelEU) and other demand-pulling policy frameworks (l. 211-217). These mechanisms remain highly uncertain given current DAC costs, which affects its competitiveness on these markets, but represent the most likely drivers for large-scale DAC deployment.

Comment 4

In conclusion, I see two ways for this paper to go: either change the focus and apply the methodology of paper #1 to a more straightforward concept of carbon capture (CCU) where you would be able to justify the use of SI_Table 5 values, or if you want to stick to DAC (and use paper #2 analogue) do it in a correct way and bring in deeper discussions on the viability of carbon markets and other demand-side levers.

Response 4

We appreciate the suggestions made by the reviewer. As pointed out in the earlier responses, we decided to follow the second suggestion and engage in deeper discussion son the viability of carbon markets and other demand-side levers.

RESPONSE TO REVIEWERS

Short-term action is key for gigaton-scale Direct Air Capture by 2050

Tatjana Zurbriggen[1], Nicoletta Brazzola[1,2*], Adrian Odenweller[3], Falko Ueckerdt[3], Joeri Rogelj[4,5]

We would like to begin by sincerely thanking the reviewers for their positive feedback and for the insightful additional comments

Below, we respond point-by-point to reviewers' comment in red. Changes throughout the manuscript and SI are color-coded as follows:

Reviewer 2 – red
Reviewer 3 – blue

REVIEWER COMMENTS

Reviewer #1 (Remarks to the Author):
The authors have addressed all of my comments, and I believe the manuscript is now ready for publication, provided that it meets the editorial and formatting requirements of Nature Communications. I would like to thank the authors for their detailed and thoughtful responses. My main concerns—particularly regarding the novelty and contribution of the study, as well as the clarification of key assumptions—have been satisfactorily addressed.

Answer:
Thanks so much for the positive feedback and, again, for the insightful comments. We really appreciate the thoughts put into the initial review and thought the comments were really helpful.

Reviewer #2 (Remarks to the Author):
I find the authors' revisions, following the largely unanimous recommendations of the three reviewers, to be substantial. However, I remain concerned that the fundamental hypothesis underlying the manuscript has not been sufficiently corroborated. The usefulness of the modeling work and its results hinges on the statement that:
"[...] in the long run, technology deployment often follows a logistic curve."
The manuscript's question is even more specific: IF DAC follows the historical trajectories of "ammonia synthesis and liquefied natural gas". Not only are there potentially hundreds or thousands of technologies with differing historical scale-up patterns that could have been used to address such "what-if" scenarios, but I also believe that the study suffers from a conceptual flaw—a category mistake.
Specifically, the authors conceptualize DAC as a single, homogeneous technology, analogous to "ammonia synthesis and liquefied natural gas". Yet the fact that these diverse processes are all labeled "DAC" does not imply the existence of a technology with a unified set of physicochemical or thermodynamic mechanisms of action. This is precisely the type of error Ryle identifies as a "category mistake" in The Concept of Mind

(1949).
In practice, it is difficult to justify that fundamentally different DAC approaches—such as amine solvent capture vs. carbonate mineralization, temperature-swing adsorption vs. humidity-swing adsorption, electrochemical vs. thermal/chemical sorbents, or vacuum-swing vs. cryogenic processes—would altogether follow the same or even similar scale-up trajectories. The term DAC is a convenient abstraction, grouping heterogeneous technologies with a common purpose but vastly different mechanisms. The manuscript's assumption that DAC behaves as a single technology with a distinct, predictable scale-up trajectory is therefore unfounded and untested.
I consider the manuscript a valuable thought experiment that highlights important questions regarding DAC scale-up. At the same time, I am not fully convinced that its current approach and assumptions justify publication in a high-profile, peer-reviewed outlet.

Answer:
We thank the reviewer for this thoughtful and philosophically grounded critique. We appreciate both the acknowledgment of the manuscript as a valuable thought experiment and the opportunity to clarify and strengthen the conceptual foundations of our modeling approach.

We agree with the reviewer that the usefulness of our modeling framework hinges on the assumption that, in the long run, technology deployment often follows a logistic (S-shaped) diffusion pattern. We would like to underline that this assumption is not ad hoc, but reflects a well-established empirical regularity in the literature on innovation diffusion, dating back at least to Rogers' seminal work *Diffusion of Innovations* (1962). Several examples, like Grübler et al.'s *Dynamics of energy technologies and global change* (1999) have since applied Rogers' theory of innovation diffusion to green technologies. Thus, the observation that many technologies exhibit S-shaped adoption trajectories is widely regarded as a scientific consensus and has been repeatedly documented across energy, industrial, and infrastructure technologies.

At the same time, we fully acknowledge—both here and explicitly in the manuscript—that projections based on logistic diffusion are inherently uncertain, especially for early-stage technologies such as DAC. Our intention is therefore not to make a deterministic claim about DAC's future deployment path, but to explore plausible *what-if* trajectories under clearly stated assumptions. To mitigate overconfidence associated with any single diffusion assumption, we deliberately adopt a fully stochastic framework, sample across uncertain initial conditions, and span a wide range of potential 2050 capacity levels. In this sense, our approach is more uncertainty-aware than much of the existing literature applying logistic diffusion models to DAC and other emerging energy technologies.

The reviewer raises two additional, closely related concerns: (i) that many alternative historical analogues could have been chosen, and (ii) that treating DAC as a single technology constitutes a category mistake in the sense articulated by Ryle (1949). We respectfully disagree with the latter characterization, while agreeing with the underlying concern regarding technological heterogeneity.

In regard to the choice of technological analogs, we use a range of technological analogues selected on the basis of relevant technology characteristics, following an established analogue-based scenario methodology (e.g., Roberts and Nemet, 2024; Nemet et al., 2023; Edwards et al., 2024). As clarified in the previous revision, “to span a broad range of uncertainty, we selected three technological analogs that share technology similarities with different types of DAC and that diverge in their historical growth rates.” This includes ammonia synthesis as an analogue for high-temperature liquid-solvent DAC, while explicitly rejecting this analogy for low-temperature solid-sorbent DAC and instead including wind energy as a closer analogue due to its modularity and mass-manufacturing potential. This reflects the fact that we are well aware of potential differences between DAC technologies.

With respect to the alleged category mistake in modeling DAC, we fully agree that DAC is not a single, homogeneous technology and that fundamentally different capture approaches (e.g., liquid solvents, solid sorbents, mineralization, electrochemical routes) may exhibit distinct learning and scale-up dynamics. However, our analysis does not assume that DAC is a single monolithic technology with an intrinsically well-defined diffusion trajectory. Nor do we claim that all DAC approaches will necessarily scale in the same way. Rather, we model *aggregate DAC deployment* while explicitly spanning uncertainty through the use of multiple, deliberately diverse technological analogues. While we could have, in principle, deployed different growth rates for distinct types of DAC and model their individual diffusion trajectories based on their initial capacity, we actively decided against this to avoid inserting further sources of uncertainties. In fact, from an innovation-dynamics perspective, literature suggests that many technologies initially evolve through competing designs before converging—partially or fully—toward dominant configurations (e.g., Anderson & Tushman, 1990; Lee et al., 1995). We agree with the reviewer that no dominant DAC design exists today, but for this exact reason it would have been hard to predict which DAC technologies would eventually be able to sink energy costs, which are the major driver of DAC competitiveness, and conquer the largest market share.

Moreover, our alleged categorical mistake would have applied to multiple previous studies of technology diffusion. Many widely studied “technologies” are in practice umbrella categories encompassing diverse designs and mechanisms. Examples include solar photovoltaics (crystalline silicon, thin-film, emerging perovskites), hydrogen electrolysis (alkaline, PEM, SOEC), or synthetic fuels (Fischer–Tropsch, methanol pathways). In all these cases, the absence of a single dominant design—especially at early stages—has not precluded meaningful analysis of aggregate deployment trajectories. Solar PV, in particular, illustrates that heterogeneous underlying technologies can nevertheless exhibit coherent aggregate diffusion patterns.

Finally, the reviewer is correct that, by design, our projections cannot be empirically tested at present. In the previous revision, we have explicitly acknowledged this limitation and emphasized that our study does not aim to forecast DAC deployment, but to explore conditional scenarios. We therefore (i) rely on analogue-based reasoning grounded in established innovation diffusion literature and (ii) clearly frame the analysis as a *what-if* exercise. This approach is consistent with prior work on emerging energy technologies and is intended to inform discussion rather than to provide definitive predictions.

We acknowledge, however, that currently the manuscript does not discuss in depth the different technologies understood under the umbrella term of DAC. In response to the reviewer's comment, we have thus further strengthened the manuscript by describing the different technologies that are often understood as 'DAC' (l. 21-23).

We hope these clarifications address the reviewer's concerna and make clear that our study does not seek to predict how DAC will scale, but rather explores whether historically observed diffusion patterns—across a deliberately wide uncertainty range—could plausibly accommodate future DAC deployment needs.

Reviewer #3 (Remarks to the Author):
Thanks for revising the manuscript and addressing mine and other reviewers' comments.
I think the paper is much improved and its limitations are well explained.
I have only one request and one minor comment:

1- It would be essential to state clearly that this analysis is not based on any techno-economic assessment of DAC technologies, nor does it consider any specific mitigation policy, R&D investment choices, or any other interactions among mitigation options.

2-a minor edit in line 199 is needed: "In [the] absence of serious...".

Answer:
We thank the reviewer for the positive feedback and reception of our revisions. We have revised according to the suggestions (l. 53-55, 171).
